# Rapid formulation of a genetically diverse phage cocktail targeting uropathogenic *Escherichia coli* infections using the UTI89 model

**DOI:** 10.1038/s41598-025-96561-y

**Published:** 2025-04-14

**Authors:** Pattida Kongsomboonchoke, Panupon Mongkolkarvin, Patiphan Khunti, Jarukit Vijitphichiankul, Poochit Nonejuie, Parameth Thiennimitr, Vorrapon Chaikeeratisak

**Affiliations:** 1 Biotechnology program, Faculty of Science, Chulalongkorn University, Bangkok, Thailand; 2 Department of Microbiology, Faculty of Medicine, Chiang Mai University, Chiang Mai, Thailand; 3 Department of Biochemistry, Faculty of Science, Chulalongkorn University, Bangkok, Thailand; 4 Center for Advanced Therapeutics, Institute of Molecular Biosciences, Mahidol University, Nakhon Pathom, Thailand; 5 Center of Excellence in Microbial Diversity and Sustainable Utilization, Chiang Mai University, Chiang Mai, 50200 Thailand

**Keywords:** Uropathogenic *Escherichia coli* (UPEC), Urinary tract infection (UTI), Multidrug-resistant (MDR) bacteria, Phage therapy, Phage cocktail, drug resistance, disease-free and overall survival, Bacteriophages, Phage biology

## Abstract

Urinary tract infections are commonly caused by uropathogenic *Escherichia coli* (UPEC). Due to the emergence of multidrug-resistant UPEC, rendering antibiotic treatment ineffective, phage combination-based therapy has been proposed as a potential alternative. Here, we present a formulation of a genetically diverse phage-derived cocktail that is rapidly customized for UPEC using *E. coli* UTI89 as a model strain. Through our rapid selection and combination of four phages against UPEC strain UTI89 (SR01, SR02, SR04, and Zappy) from our library, the combination of two lytic phages, SR02 and SR04, exhibits the strongest suppression of bacterial growth for at least 16 h, with no emergence of phage resistance observed in vitro. Phage SR02 undergoes subcellular activity for 25 min, producing approximately 106 progeny particles per cell, while SR04 completes its replication cycle in 20 min, generating around 564 progeny particles per cell. These two novel phages are genetically diverse, and their cocktail exhibited potent suppression of bacterial growth, independent of multiplicities of infection (MOIs), significantly reducing the viable bacterial counts after treatment in vitro. The phage cocktail has low immunogenicity and does not induce any proinflammatory gene responses in human bladder uroepithelial cells. Moreover, the cocktail effectively eradicates the invading UPEC strain UTI89 in the uroepithelial cells at a comparable level to that of phage SR04 alone, likely releasing some immunostimulatory agents that, in turn, trigger upregulation of *MIP-3* and *IL-8* genes. Altogether, this study offers an alternative pipeline for rapidly formulating genetically diverse phage-derived cocktails, which is specifically customized for targeted bacteria.

**Supplementary Information:**

The online version contains supplementary material available at 10.1038/s41598-025-96561-y.

## Introduction

Urinary tract infections (UTIs) are significant global health concerns, affecting over 404.6 million people and resulting in nearly 240,000 deaths annually^1^. The infections are caused by uropathogens, including both Gram-negative and Gram-positive bacteria, as well as some fungi, with uropathogenic *Escherichia coli* (UPEC) being the most commonly identified causative pathogen^2^. UPEC firstly colonizes the urethra and urinary bladder, leading to cystitis. The infection can then progress by ascending through the ureters to colonize the kidneys, resulting in pyelonephritis^3^. After invading the urothelial cells, it proliferates and forms clusters within the cytosol. These clusters are encased in biofilms, providing protection from the immune response and the surrounding environment^4^. Antibiotic treatment regimens of UTIs are typically used to manage the infection and are based on the identification of the infection, bacterial characterization, and antimicrobial resistance profiles^5^. However, the misuse and overuse of antibiotics have accelerated the emergence of multidrug-resistant (MDR)-UPEC, including the resistance to ampicillin, cephalosporins, fluoroquinolones, aminoglycosides, and trimethoprim-sulfamethoxazole^4–8^. Moreover, certain UPEC strains have been found to produce extended-spectrum β-lactamases (ESBLs), which function in inactivating β-lactam antibiotics^9^. The World Health Organization (WHO) has classified ESBL-producing  *Enterobacterales* (including UPEC) as one of the critical groups of drug-resistant bacteria in urgent need of new effective treatments^10^. Therefore, research and development of novel effective therapeutic strategies for infectious disease caused by UPEC are urgently needed.

Bacteriophages or phages are bacterial viruses with simple structures, consisting of DNA or RNA-enclosed within a protein capsid. They are highly diverse, more than bacteria, as they act as bacterial parasites^11^. Phages can be classified into temperate (lysogenic) and virulent (lytic) phages based on their life cycles^12,13^. Phage therapy has been proven to be highly effective in treating drug-resistant bacterial infections, providing satisfactory results in urgent cases, such as infections caused by drug-resistant *Mycobacterium abscessus*, *Acinetobacter baumannii*, and *Pseudomonas aeruginosa*^14–16^. Several studies have reported novel phages that effectively target and inhibit the growth of UPEC, offering promising alternatives to antibiotic treatment^17–23^. For example, phage vB_EcoP-EG1 was able to eliminate biofilms formed by *E. coli* MG1655 and specifically targeted 10 out of 21 clinical MDR-UPEC strains^18^. Phage VB_EcoS-Golestan demonstrated suitable therapeutic characteristics, including rapid adsorption, large burst size, and effective inhibition of UPEC growth^22^. Phages ϕEc1 and ϕEc3 showed the ability to reduce biofilm formation and bacterial adherence^17^. MLP phages not only effectively killed UPEC but also demonstrated the ability to infect enteroaggregative *E. coli*^21^. Two lytic phages, HP3 and ES17, reduced the fitness of phage-resistant UPEC in pooled human urine, potentially leading to increased host immunity or antibiotic susceptibility^23^. The synergistic effects of phage and antibiotic treatments were confirmed in many studies involving phages FS2B and Killian, suggesting the potential for future development of phage-antibiotic combination therapies^19,20^.

Nevertheless, a notable limitation of phage therapy is the restricted depth of a phage’s spectrum of activity, often due to the rapid development of phage resistance in targeted bacterial isolates^24^. Like antibiotics, the need for strategies to overcome this resistance is urgently needed. One promising approach is through the use of combinations of multiple phages in treatments, which has been proven to enhance therapeutic efficacy^25–27^. Recently, we demonstrated that the careful selection of phages for combination is critical, as combining genetically diverse phages in a cocktail can significantly enhance bacterial suppression and delay the development of phage resistance^28^. Building on this principle, we hypothesized that selecting phages for formulations based on their superior efficacy once combined would guide us towards identifying genetically diverse phages. In this proof-of-concept study, we used Uropathogenic *E. coli* isolate UTI89, previously obtained from a patient with an acute bladder infection^29^, as the bacterial host model. Through rapidly screening and combining phages from our library against the bacteria, we identified two phages, designated SR02 and SR04, as optimal candidates for cocktail formulation. We further characterized the morphological, biological, and genomic features of these selected phages to assess their genetic diversity and suitability for therapeutic use. When combined, these genetically diverse phages demonstrated a strong enhancement in suppressing *E. coli* UTI89 growth and significantly prolonged the time to bacterial regrowth in vitro. We lastly demonstrated the prophylactic potential of this formulated phage cocktail against *E. coli* UTI89 infection in a human bladder uroepithelial cell culture model.

## Results

### Phage discovery and selection of virulent phages

To obtain phages targeting the bacterial host, uropathogenic *E. coli* (UPEC) strain UTI89, the bacterial model in this study, was used to enrich phages from samples collected from canals in Bangkok, Thailand. *E. coli* UTI89 is a well-characterized cystitis isolate, with both genotypic and phenotypic characteristics, and also clinical pathogenicity^30–36^. Hence, it is suitable for studying the interaction with novel treatments. Through the selection of individual phages based on plaque morphology displayed on Double layer agar (DLA) plates (Supplementary Fig. S1), we found that plaques of SR01 and SR04 seemed similar to SR02 and Zappy, respectively. Specifically, phages SR01 and SR02 produced small, turbid plaques with diameters of less than 1 mm, whereas SR04 and Zappy produced clear plaques with larger diameters exceeding 3 mm. Therefore, restriction fragment length polymorphism (RFLP) analysis for those with indistinguishable plaque morphologies was performed to confirm that they were distinctive. The results of phage genomes cut with the enzyme *Eco*RV and its combination with *Eco*RI in Supplementary Fig. S2 show that SR01 and SR02 can be differentiated based on unique DNA fragment patterns when cut with *Eco*RV alone. Similarly, once cut with the combination of *Eco*RI and *Eco*RV, the differences pattern between the cut genomes of SR04 and Zappy were revealed. These data confirm the successful isolation of four unique phages, designated as SR01, SR02, SR04, and Zappy.

**Fig. 1:**
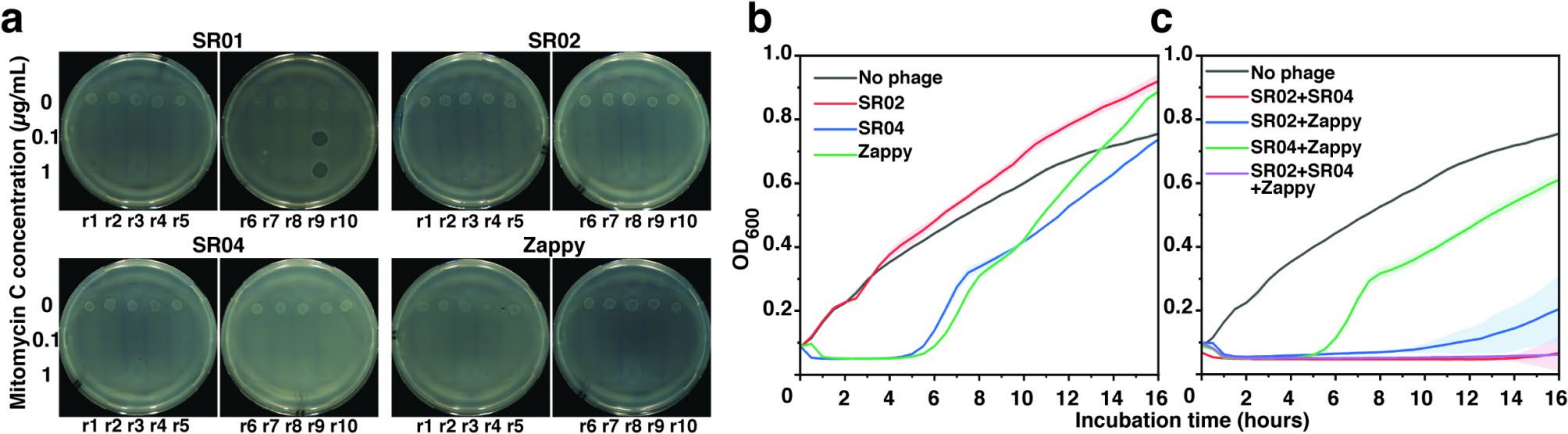
Rapid phage selection to formulate an efficient phage cocktail against uropathogenic *E. coli* UTI89. **(a)** Lysogeny test of isolated phages; SR01, SR02, SR04, and Zappy, by prophage induction using mitomycin C treatment at 0.1 and 1 µg/mL. Phage-resistant isolates from the corresponding phage as indicated on the top of the panels were named as r1-r10. Filtrated liquid cultures of mitomycin C treated phage-resistant isolates were dropped on the bacterial lawn on DLA plates and the presence of plaques indicates phages releasing from lysogens. Killing assay based on bacterial cell density during a window of 16 h-incubation times of high titer of **(b)** individual phages and **(c)** phage combinations (~ 10^8^ PFU/mL). Experiments in **(b)** and **(c)** were conducted in three biological replicates and data were shown as the mean with standard deviation.

To ensure the safety of these phages for therapeutic use, we first aimed to eliminate phages capable of integrating their genome into the host chromosome. Phages were examined for temperate characteristics through prophage induction using mitomycin C in resistant isolates. Upon mitomycin C treatment, the control bacterial host UTI89 was not lysogenized by any phages and thus can be used for raising phage-resistant isolates (Supplementary Fig. S3; negative control). We then raised phage-resistant isolates against SR01, SR02, SR04, and Zappy, followed by the preparation of individual resistant-bacteria cultures (designated r1 to r10) (Fig. 1a). These resistant isolates (r1 to r10) raised from individual phages were subsequently treated with mitomycin C at 0.1 µg/mL and 1 µg/mL to induce the lytic cycle in prophages, if present. The results showed that plaques were observed from the culture of SR01-resistant isolates clone 9 (r9), when treated with mitomycin C (Fig. 1a; SR01), indicating that r9 contains the prophage of SR01. Since SR01 is a temperate phage, it was discarded from our selection. No plaques were observed from other treated phage-resistant isolates (Fig. 1a; SR02, SR04, and Zappy), suggesting that phages SR02, SR04, and Zappy are likely lytic phages and could be suitable for phage therapy.

### Rapid phage combination leads to the formulation of a phage cocktail, effectively suppressing bacterial growth and prolonging phage resistance emergence

To rapidly evaluate the killing efficacy of the selected phages against *E. coli* UTI89, we used high-titer phages (approximately 10^8^ PFU/mL) in treating the bacteria and monitored cell density for 16 h. The result revealed that SR04 and Zappy displayed similar killing patterns in suppression of bacterial growth for at least 5 h, during which OD_600_ remained below 0.05. However, an increase in cell density was observed, reaching an OD_600_ of approximately 0.35 at 7–8 hours, followed by a gradual increase to level comparable to the untreated control by the end of the experiment (Fig. 1b; blue and green lines), suggesting the possible emergence of phage-resistant bacteria in the population. However, SR02 did not show an inhibitory effect on bacterial growth during the incubation period (Fig. 1b; red lines). Since phage resistance typically arises during single-phage therapy, and combining phages can delay this resistance^37^, we then combined the selected phages to create all possible phage cocktail formulas (Fig. 1c): SR02 + SR04, SR02 + Zappy, SR04 + Zappy, and all phages. As expected, the combination of all three phages exhibited the best outcome, entirely suppressing bacterial growth throughout the experimental period (Fig. 1c; purple line). Intriguingly, the combination of SR02 and SR04 displayed the same pattern of bacterial suppression as the combination of all three phages, suggesting that Zappy does not play a significant role in bacterial killing or minimizing phage resistance when SR02 and SR04 are present (Fig. 1c; red line). This observation was further confirmed when Zappy was combined with either SR02 or SR04, as bacterial regrowth was observed during late treatment: after about 7 h when mixed with SR02 (Fig. 1c; blue line) and after about 5 h when mixed with SR04 (Fig. 1c; green line). Given that the combination of SR02 and SR04 alone provided comparable outcomes to the three-phage combination (SR02, SR04, and Zappy), Zappy was deemed unnecessary for bacterial suppression. We also observed varying degrees of bacterial suppression across different phage combinations. Based on our previous study, which demonstrated that genetically diverse phage combinations enhance cocktail efficacy^28^, along with the data shown in Fig. 1c, Supplementary Figure S1, and S2 (plaque morphology and RFLP analysis), we hypothesize that the superior bacterial suppression observed in the combination of SR02 and SR04 may be partly attributed to their genetic diversity. This hypothesis is further supported by the strong inhibitory effect of the SR02 + Zappy combination (Fig. 1c and Supplementary Fig. S1), as these phages, similar to SR02 and SR04, displayed distinct plaque morphologies indicative of genetic divergence. In contrast, the combination of SR04 and Zappy displayed reduced effectiveness compared to other combinations (Fig. 1c). This finding also strengthens our hypothesis, as the combination of these genetically closely related phages (Supplementary Fig. S1 and S2) may limit the overall potency of the cocktail. Altogether, these observations highlight the role of genetic diversity among phages in enhancing their antibacterial efficiency. Consequently, we selected phages SR02 and SR04 for subsequent experiments to further investigate this hypothesis.

### Host range determination and lysis profile

To further elucidate the host range of all phages and determine their potential against multiple bacterial strains, a host specificity assay was conducted against 13 distinct *E. coli* strains, including clinical UPEC isolates, and 1 *Salmonella* spp. These *E. coli* strains consisted of commensal, probiotic, uropathogenic, and diarrheagenic isolates. Apart from our *E. coli* UTI89 as a model, UPEC strains include *E. coli* CFT073, a pyelonephritis isolate^38^, and *E. coli* ABU83972, an asymptomatic strain^39^. *E. coli* ATCC25922 and *E. coli* MC4100 represent commensal strains, commonly used as laboratory models due to their extensive characterization^40–42^. *E. coli* Nissle 1917, a well-studied probiotic strain^43^, was also tested. More information of individual lab and reference strains is presented in Table 1 and Supplementary Table S1. To also evaluate the spectrum of phage activity in clinical settings, 6 clinical isolates that were randomly selected from different acute cystitic pateints at different times in 2020 at Maharaj Nakorn Chiang Mai Hospital, Chiang Mai, Thailand were included (Table 1 and Supplementary Table S2). Despite the diversity of these distinct bacterial groups, all phages were highly specific to *E. coli* UTI89 and exhibited a relatively restricted host range (Table 1). Among the tested phages, SR02 exhibited the broadest host range, successfully infecting 4 out of 14 strains. Specifically, SR02 displays EOP for UTI89, CFT073, ATCC25922, and UPEC AT3 at 1, 0.37, 0.3, and < 0.001, respectively. In contrast, SR04 and Zappy displayed identical host specificity and a narrow host spectrum infecting their parental host at EOP equal to 1 and UPEC AT4 at EOP below 0.001. Along with our finding aforementioned and since SR01 is a temperate phage and Zappy does not contribute to expanding the host range, only the phages SR02 and SR04 were selected for subsequent experiments.

**Table 1:** Efficiency of plating (EOP) of phages SR01, SR02, SR04, and Zappy. Values were classified as highly productive (≥ 0.5), medium productive (0.1 ≤ EOP < 0.5), low productive (0.001 < EOP < 0.1), or inefficient (≤ 0.001). The hyphen (-) refers to an inability of phages to lyse the bacterial isolates. Additional data on bacterial isolates are provided in table S1 and S2.

Bacterial strains and sequence type (ST)		Sources (place and year of isolation)	Phages			
			SR01	SR02	SR04	Zappy
Symptomatic UPEC strains	*E. coli* UTI89 (ST95 ^96^)	A patient with acute cystitis (United States, 2000) ^97^	1	1	1	1
	*E. coli* CFT073 (ATCC 700928) (ST73 ^98^)	The blood and urine of a woman with acute pyelonephritis (Baltimore, Maryland, United States, 1990) ^38^	-	0.37	-	-
Asymptomatic UPEC strains	*E. coli* ABU83972 (ST73 ^99^)	The urine of a young girl with a three-year history of asymptomatic bacteriuria (ABU) with stable renal function (Gothenburg, Sweden, 1975) ^100^	-	-	-	-
Commensal *E. coli* strains	*E. coli* ATCC25922	A clinical sample with multiple antibiotic resistance profiles (Seattle, Washington, United States, 1976) ^101^	-	0.3	-	-
	*E. coli* MC4100	A *lac* deletion strain from *E. coli* K-12 strain MG1655 (Boston, United States, 1976) ^102^	-	-	-	-
Probiotic *E. coli* strains	*E. coli* Nissle 1917	A soldier with severe of diarrhea during WWI (Germany, 1917) ^103^	-	-	-	-
Clinical UPEC isolates	UPEC AT1	Midstream urine culture from a cystitic 67-year-old female patient (Chiang Mai, Thailand, 9/26/2020)	-	-	-	-
	UPEC AT2	Midstream urine culture from a cystitic 81-year-old male patient (Chiang Mai, Thailand, 8/8/2020)	-	-	-	-
	UPEC AT3	Midstream urine culture from a cystitic 67-year-old male patient (Chiang Mai, Thailand, 10/9/2020)	-	< 0.001	-	-
	UPEC AT4	Midstream urine culture from a cystitic 41-year-old female patient (Chiang Mai, Thailand, 9/24/2020)	-	-	< 0.001	< 0.001
	UPEC AT5	Midstream urine culture from a cystitic 74-year-old female patient (Chiang Mai, Thailand, 9/26/2020)	-	-	-	-
	UPEC AT6	Intermittent catheterized urine culture from a cystitic 74-year-old female patient (Chiang Mai, Thailand, 1/10/2020)	-	-	-	-
Diarrheagenic *E. coli* strain	Enterotoxigenic *E. coli* (ATCC 35401)	Feces of individuals with acute non-specific diarrhea (West Pakistan, 1972) ^104^	-	-	-	-
*Salmonella enterica* Typhimurium	Nalidixic acid-resistant derivative of ATCC 14028	The pooled heart and liver tissue of 4-week-old chickens (France, 1987) ^105^	-	-	-	-

### Morphological studies of SR02 and SR04

**Fig. 2:**
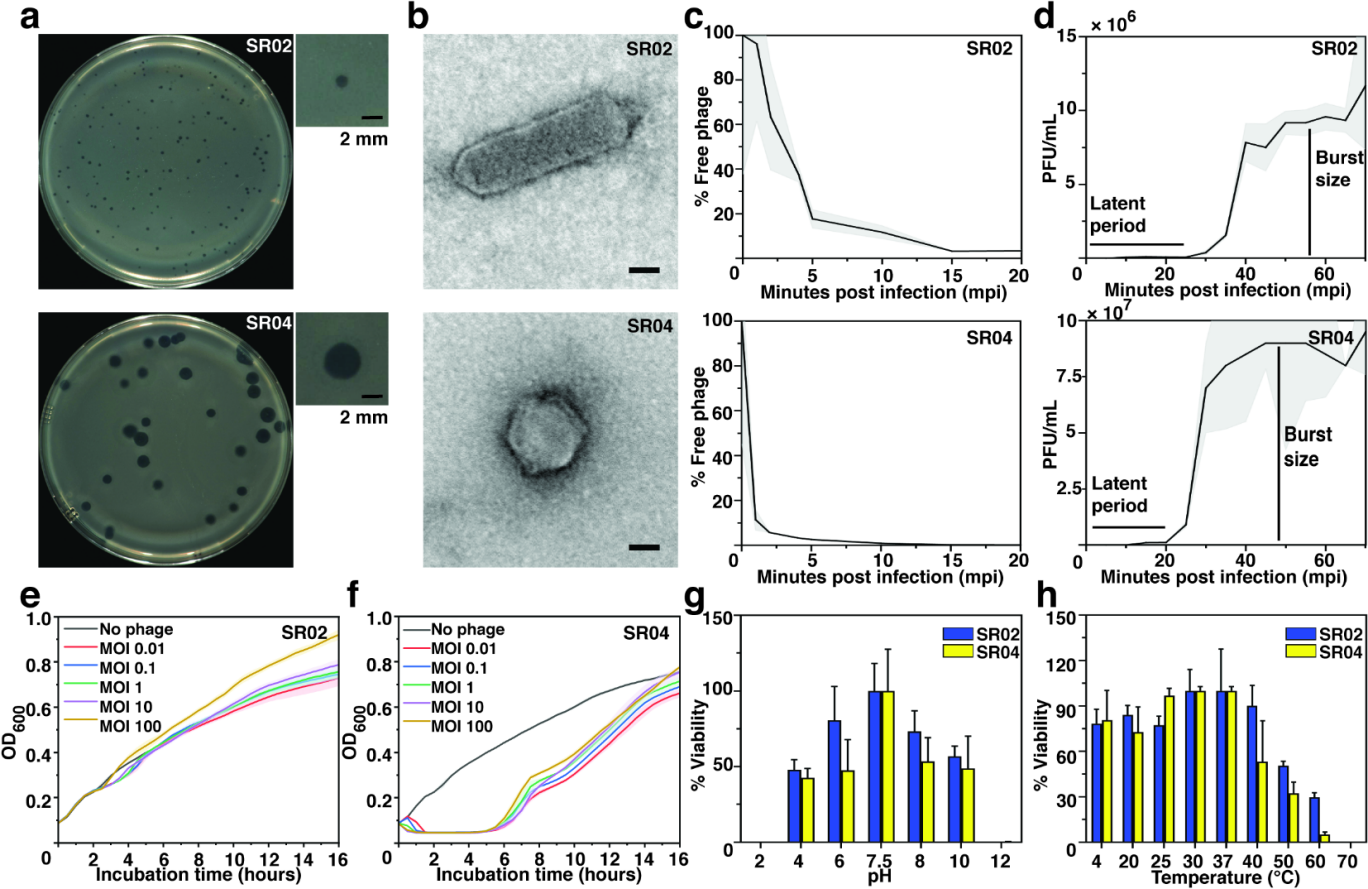
Morphological and biological properties of selected phages SR02 and SR04. **(a)** Plaque morphology of phages SR02 (upper panel) and SR04 (lower panel) on the bacterial cell lawn of *E. coli* UTI89. An individual plaque of each phage is enlarged as shown in the top right panel, next to each image. Scale bars equal 2 mm. **(b)** Transmission electron microscope (TEM) images of purified phages SR02 (upper panel) and SR04 (lower panel). Scale bars equal 20 nm. **(c)** Adsorption assay of SR02 (upper panel) and SR04 (lower panel). **(d)** One-step growth curve of SR02 (upper panel) and SR04 (lower panel). Killing assay of **(e)** SR02 and **(f)** SR04 at different MOIs ranging from 0.01 to 100. Tolerance test of phages at various pHs **(g)** and temperatures **(h)**. **(c-h)** Experiments were conducted in at least three biological replicates and data were shown as the mean with standard deviation.

To further explore the characteristics of the selected phages, SR02 and SR04, morphological studies were conducted. As revealed by DLA plates of *E. coli* UTI89 lawn, SR02 produced turbid, variable-sized plaques with an average diameter of 0.81 ± 0.25 mm (mean ± SD, *n* = 31), while SR04 produced smooth-edged, clear plaques with an average diameter of 3.75 ± 0.61 mm (*n* = 15) (Fig. 2a; upper and lower panels). Negative staining TEM revealed the rare C3 morphotype of SR02, characterized by an elongated capsid approximately 132.73 ± 10.52 nm in length (*n* = 11) with a short tail, and the C1 morphotype of SR04, composed of a 50.52 ± 1.52 nm (*n* = 29) icosahedral capsid and a short tail (Fig. 2b; upper and lower panels)^44,45^. These structural and plaque morphology differences confirm that SR02 and SR04 are distinct phages.

### Biological studies of SR02 and SR04

The biological properties of phages were also investigated, including adsorption assays, one-step growth curves, killing assays, and phage tolerance tests. We found that more than 80 percents of both phages were capable of rapidly adsorbing to *E. coli* UTI89 within 5 min (Fig. 2c; upper and lower panels). Although they shared similar adsorption rate, SR04 required less time (around 20 min) to propagate within host cells compared to SR02 (around 25 min) (Fig. 2d; upper and lower panels). Upon completing their replication cycle, SR04 released approximately 564 progeny particles per cell, whereas SR02 produced only around 106 particles per cell, indicating a less efficient reproduction process for SR02 in this host. This different reproductive efficiency of SR02 and SR04 was further supported by their respective killing curve against UTI89 (Fig. 2e and f). Regardless of the phage concentrations, SR04 exhibited potent killing activity, completely suppressing bacterial growth during the initial 5–6 h, even at the lowest MOI (Fig. 2f). In contrast, SR02 demonstrated only moderate inhibition, slightly slowing bacterial growth between 3 and 4 h across MOIs ranging from 0.01 to 10, but was unable to reduce bacterial density further afterward (Fig. 2e). As demonstrated by tolerance tests, both phages were quite tolerant across a wide range of pH and temperature conditions, with SR02 slightly outperforming SR04. In particular, both phages were completely inactivated at extremely acidic (pH 2) and basic (pH 12) conditions, as well as temperatures exceeding 70 °C (Fig. 2g and h).

### Genome analysis of SR02 and SR04 demonstrates their safety for therapeutic use and their genetic divergence

**Fig. 3:**
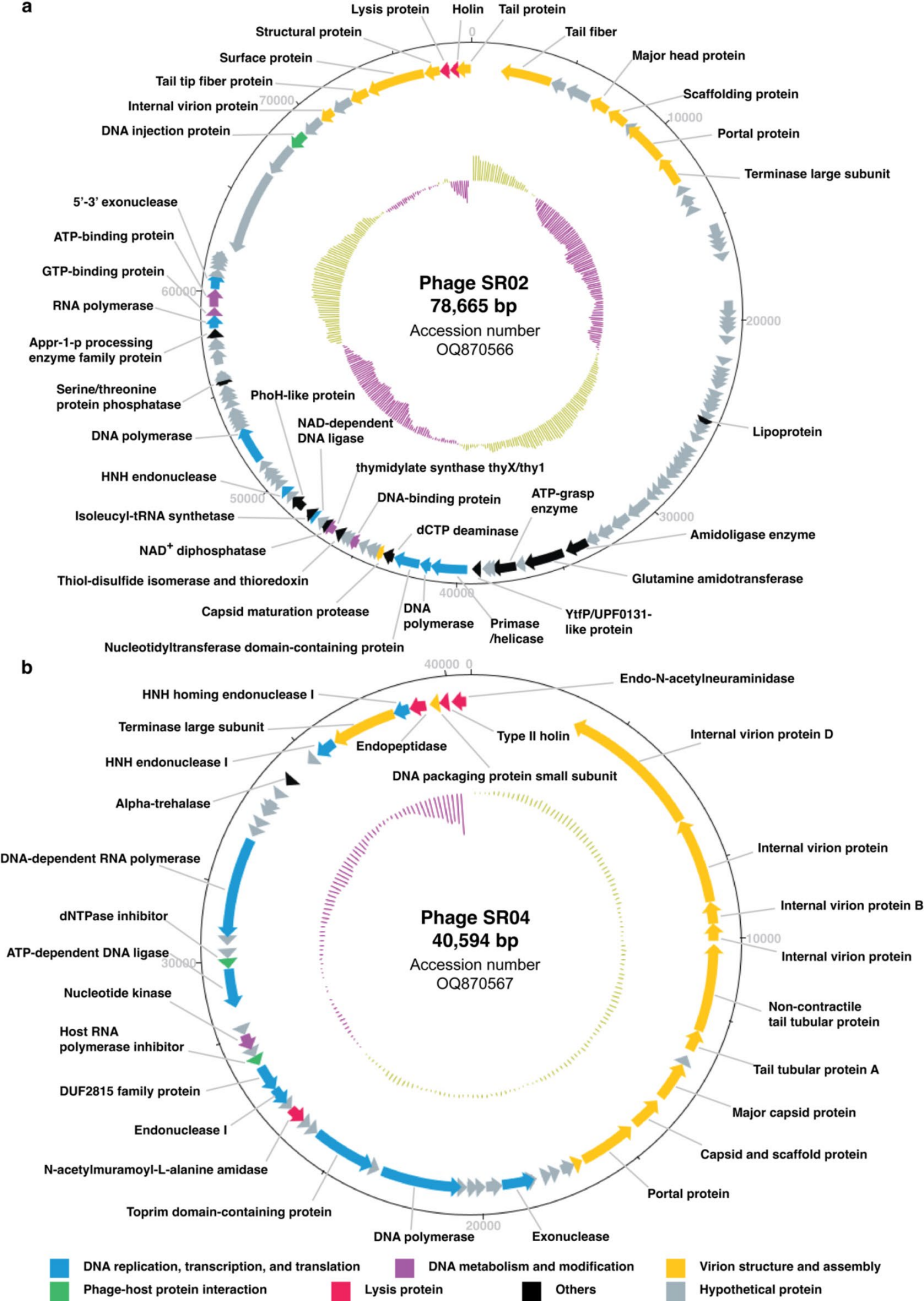
Schematic genome maps of phages SR02 (**a**) and SR04 (**b**). The open reading frames (ORFs) are indicated with arrows showing the ORF direction and the functional annotated genes are categorized into 6 different groups; DNA replication, transcription, and translation (blue), DNA metabolism and modification (purple), virion structure and assembly (yellow), phage-host protein interaction (green), lysis protein (red), and others (black). Apart from those functionally annotated genes, the rest are classified as hypothetical proteins, indicated by grey color. The innermost circles represent GC content across the genome (yellow; above average, and purple; below average). Related to Supplementary Tables S3 and S4.

In addition to the desirable characteristics of phages that contribute to their high infectivity against targeted bacteria, genetic information is crucial for selecting non-harmful, appropriate phages for therapeutic use. To ensure the absence of undesirable genes associated with antibiotic resistance, bacterial virulence, and lysogenic life cycles in the phage genomes, we performed whole-genome sequencing using Illumina MiSeq platform. *de novo* assembly was carried out, and all open reading frames (ORFs) were annotated across the phage genomes. We obtained 4,451,354 reads for SR02 and 4,096,119 reads for SR04, successfully assembling the complete genomes of SR02 and SR04 with coverage over 12,000X and 6,000X, respectively (Fig. 3). The genomes of both phages were linear, double-stranded DNA. The complete genomes of SR02 were 78,665 bp in length with a GC content of 41.9%, while SR04’s genome was 40,594 bp long with a GC content of 49.8% (Fig. 3a; accession numbers OQ870566, and Fig. 3b; accession numbers OQ870567). Structural ORFs annotated using DNA Master software version 5.0.2 (https://phagesdb.org/DNAMaster/)^46^ showed that SR02 encoded 100 ORFs in the forward direction and 23 ORFs in the reverse direction (Fig. 3a and Supplementary Table S3), while SR04 encoded 52 structural ORFs in the reverse direction (Fig. 3b and Supplementary Table S4). Among the annotated ORFs, 40 ORFs in SR02 and 33 ORFs in SR04, providing significant hits of E-value less than 10^− 5^ (Supplementary Tables S3 and S4), were identified as putative functional proteins, while the remaining ORFs were hypothetical proteins. The functional ORFs were classified into six main categories: (1) DNA replication, transcription, and translation, (2) DNA metabolism and modification, (3) virion structure and assembly, (4) phage-host protein interaction, (5) lysis protein, and (6) others. Importantly, no virulence or antimicrobial resistance gene were detected in either phage genomes, assuring their safety for therapeutic applications. Even though the SR02 genome contains attachment sites (attL and attR, Supplementary Table S3), which are involved in site-specific recombination systems^47^, no integrase gene was found, suggesting that SR02 is unlikely to integrate into the host genome. To further validate the life cycle of SR02, in addition to our lysogeny test (Fig. 1a), we employed two online web-based tools, PhageScope^48^ and Phage AI^49^, both of which confirmed that SR02 is a virulent phage with high confidence (Supplementary Fig. S4), supporting its safety for use.

**Fig. 4:**
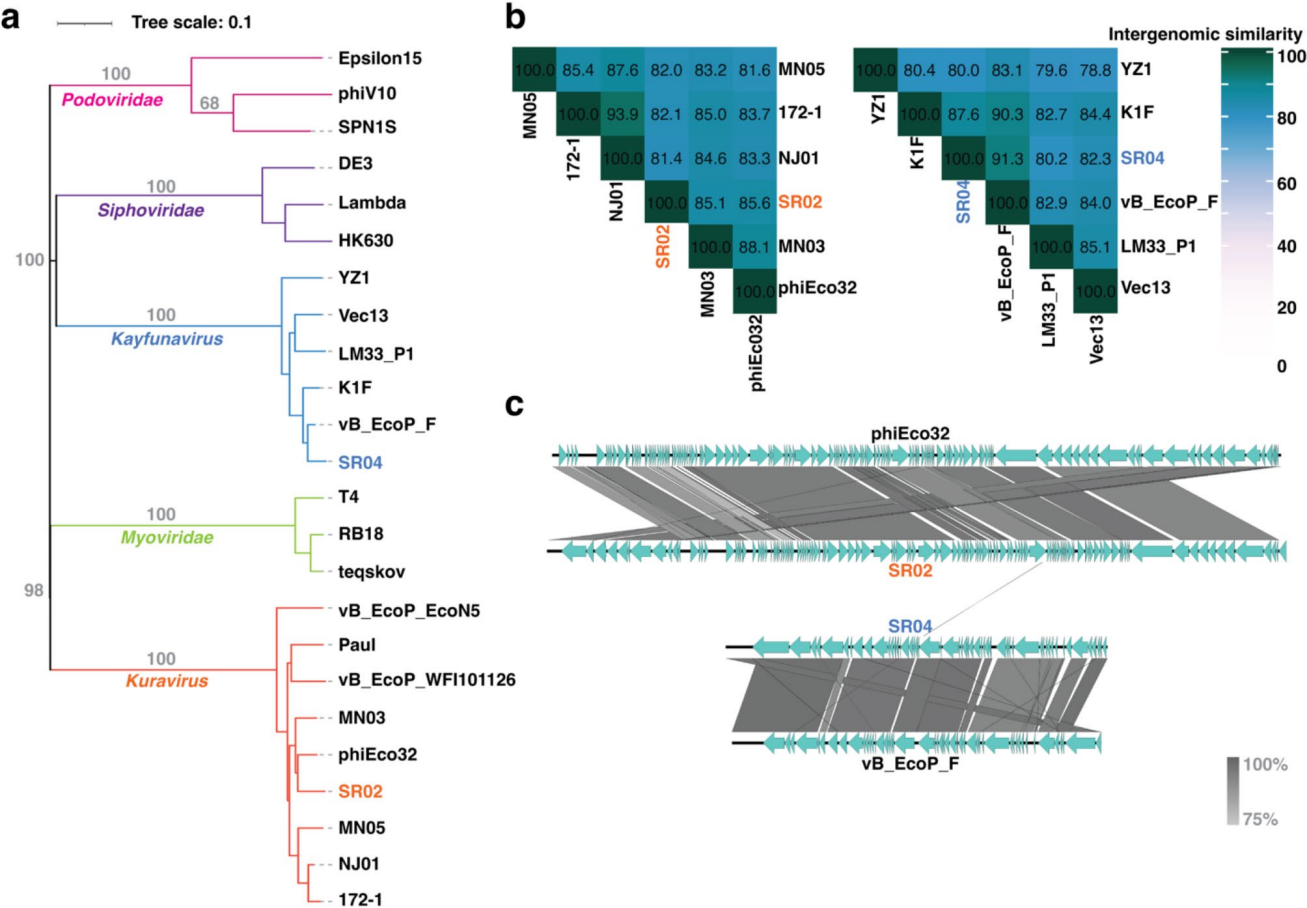
Phages SR02 and SR04 are genetically divergent and are clustered into different genera. **(a)** A whole genome-based phylogenetic tree of phages SR02 (orange) and SR04 (blue) was constructed using the Genome-BLAST Distance Phylogeny (GBDP) method and VICTOR web service with 100 bootstraps. Representative phage species are selected from the search of SR02 and SR04 against the NCBI and ICTV databases. Numbers at branches and branch colors represent the percentage of bootstrap values and phage clusters, respectively. The phage genus is indicated at the branch of each phage cluster. **(b)** VIRIDIC heatmap illustrating the intergenomic similarities of SR02 (orange) and SR04 (blue) with their closely related phages. The numbers represent the intergenomic similarity values for each genome pair. **(c)** Comparative genomic analysis of SR02 (orange) and SR04 (blue) with their closest related phages (phiEco32 and vB_EcoP_F). Green arrows represent the directions and locations of coding sequences. Gray-shaded lines reflect the degree of homology between them.

Phage cocktails composed of genetically diverse phages are more effective than those composed of phages that are closely related^28^. This is partly due to the uncommon multiple mutations in bacterial hosts that can confer resistance. We hypothesized that the high efficacy of the SR02 and SR04 combination in inhibiting bacterial growth and prolonging bacterial regrowth is due to the genetic divergence between the two phages. To explore their genetic relationship, we employed the genomes of SR02 and SR04 as queries to search against phage databases and selected closely related phages for phylogenetic analysis. Blastn search revealed that the genome of SR02 is highly similar to phages in the genus *Kuravirus*, while the genome of SR04 is closely related to phages in the genus *Kayfunavirus*. Phylogenetic analysis further revealed that SR02 and SR04 cluster separately within different clades, grouping with their closely related phages in their respective genera (Fig. 4a; orange and blue lines). To further compute the intergenomic similarity between SR02, SR04, and other related phages, VIRIDIC was performed. The results indicated that the intergenomic similarity of SR02 with phages MN05, 172-1, NJ01, MN03, and phiEco32 was 82.0, 82.1, 81.4, 85.1, and 85.6, respectively (Fig. 4b; left panel). For SR04, the intergenomic similarity with phages YZ1, K1F, vB_EcoP_F, LM33_P1, and Vec13 was 80.0, 87.6, 91.3, 80.2, and 82.3, respectively (Fig. 4b; right panel). These analyses demonstrated that SR02 and SR04 were most closely related to phiEco32 and vB_EcoP_F, respectively, but have diverged into new species, following the thresholds for the demarcation of viruses^50–52^. The genome organization of SR02 and SR04 compared to their closest related counterparts further elucidated that, despite high sequence similarity, their genes and genome organization are differently organized (Fig. 4c). Notably, we observe only one shared homolog between the genomes of SR02 and SR04, indicating their distant evolutionary relationship, which may explain the enhanced efficacy of their combination in phage cocktails, as observed in this study (Fig. 1c) and supported by previous findings^28^.

### The phage cocktail efficiently suppresses the growth of uropathogenic *E. coli* UTI89 in vitro

**Fig. 5:**
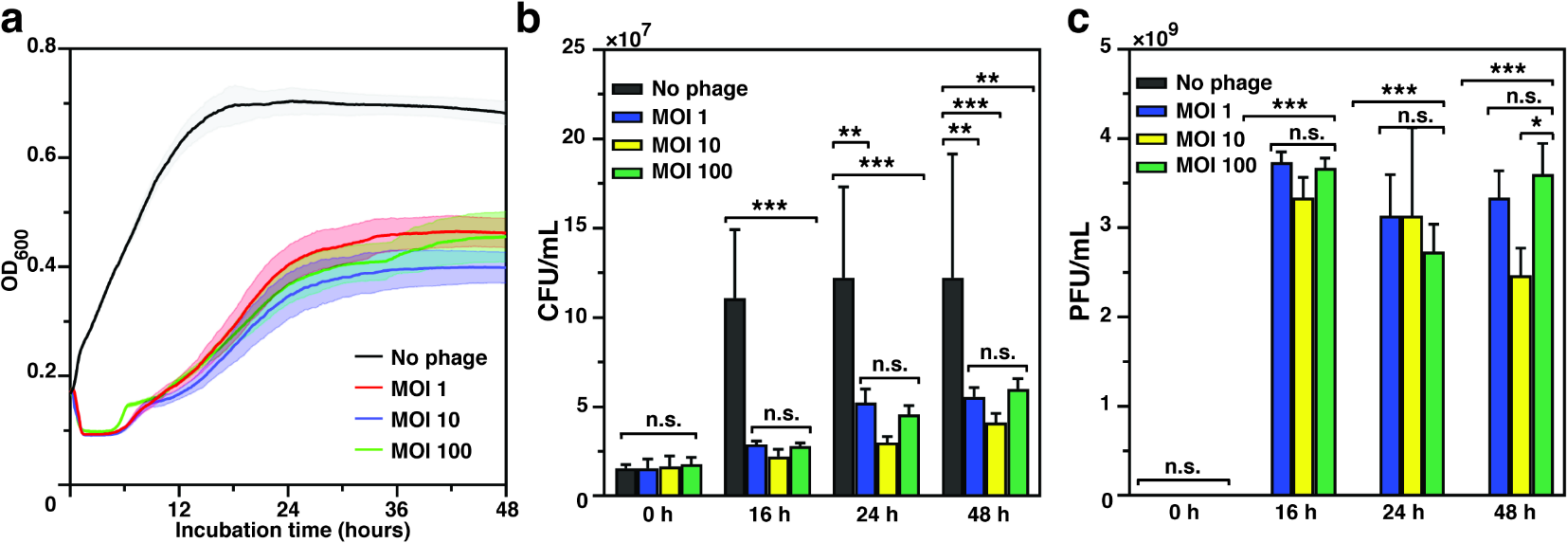
The combination of phages SR02 and SR04 efficiently suppresses the growth of uropathogenic *E. coli* UTI89 in vitro. **(a)** Killing curve of the phage cocktail at different multiplicity of infections (MOIs), ranging from 1–100, against uropathogenic *E. coli* UTI89 during 48 h of incubation. The concentration of **(b)** surviving bacteria and **(c)** viable phages at 0, 16, 24, and 48 h during the phage cocktail treatment. The data shown in **(a-c)** represent the mean with a standard deviation of at least three biological replicates. According to one-way ANOVA followed by Tukey’s HSD post hoc test, asterisks represent the p-value (**p < 0.01 and ***p < 0.001) and n.s. represents a non-significant difference.

To carefully evaluate the efficiency of the phage combination of genetically diverse phages SR02 and SR04, we first assessed the killing efficiency of the two phages in equal ratios, using MOIs ranging from 1 to 100, against the bacterial host *E. coli* UTI89 in vitro. Bacterial cell density in each condition was monitored over a 48-hour period, compared to the condition without phages as a control. The result revealed the phage cocktail exhibited potent activity in suppressing bacterial growth during the first 12 h of incubation, regardless of the MOIs used. However, an increase in bacterial density was observed after 13 h of incubation. Despite this revival, the stationary phase culture of *E. coli* UTI89 in the presence of phages showed a lower cell density compared to the condition without phage treatment (Fig. 5a). We further evaluated the number of remaining bacteria that survived the treatment at 16, 24 and 48 h (Fig. 5b). Consistent with the bacterial suppression profile, the phage combination significantly reduced bacterial numbers by approximately 4-fold and 3-fold at 16 and 24 h, respectively, compared to the control (absence of phages), indicating the high potency of the phage cocktail (Fig. 5b; 16 and 24 h). Additionally, although bacterial growth remained significantly suppressed at 48 h in the presence of phages, bacterial numbers appeared to slightly increase compared to previous time points (Fig. 5b; 16, 24, and 48 h). This observation suggests the emergence of phage-resistant bacterial populations during prolonged treatment. Notably, no significant difference in bacterial numbers across MOIs was observed at any time point. To assess whether viable phages remained present throughout the experiment, we also quantified the viable phages during treatment. We found that phage concentrations remained relatively high, exceeding 2 × 10^9^ PFU/mL throughout the incubation (Fig. 5c; 16 and 24, and 48 h). These data suggest that the phage cocktail effectively suppresses the growth of *E. coli* UTI89, but its potency may be compromised during prolonged treatment as some phage-resistant bacteria may arise to overcome the phage infection.

### The phage cocktail reduced the invasion of *E. coli* UTI89 into human bladder urothelial cells and altered the expression of certain proinflammatory cytokine genes

**Fig. 6:**
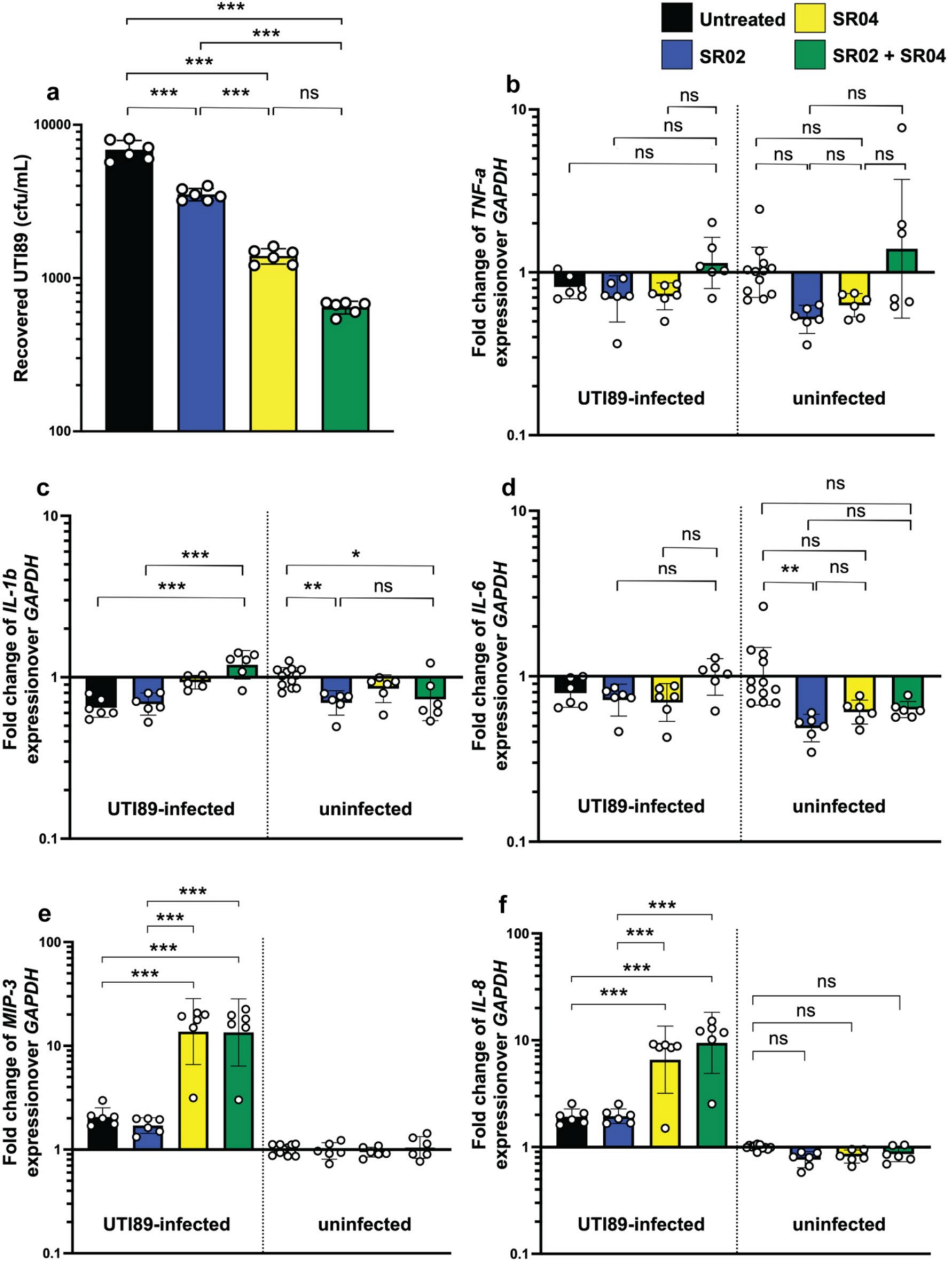
The therapeutic aspect of the phage cocktail. Phages SR02 and SR04 reduced the invasion of uropathogenic *E. coli* (UPEC) and increased some proinflammatory cytokine gene expressions in human bladder urothelial cells. Human bladder epithelial cells (UM-UC-3) (10^5^ cells per well) were pretreated with phage SR02, SR04, or the combination (MOI = 20) for five minutes before subsequent UPEC infection (100 µL of 10^8^ CFU/mL) for 1 h. The recovered *E. coli* UTI89 numbers in each group were enumerated **(a)**, and expression of proinflammatory cytokine genes TNF-a, IL-1b, IL-6, MIP-3, and IL-8 from UM-UC3 were measured **(b**,** c**,** d**,** e**, and** f**, respectively). Bars represent the geometric mean, while a geometric standard deviation is shown by error bars. *, **, *** indicate P-value < 0.05, 0.01, and 0.001, respectively, ns, a non-significant difference.

A gentamicin protection assay was used to determine whether phages SR02, SR04 or the cocktail could reduce the invasiveness of *E. coli* UTI89 on human bladder urothelial UM-UC-3 cells^53,54^. Our data showed that pre-treatment of UM-UC-3 cells with phages SR02, SR04, or the cocktail significantly reduced the numbers of invading *E. coli* UTI89 by approximately 2-, 5-, and 11-fold, respectively, compared to untreated cells (Fig. 6a), with phage SR04 alone presented a significantly greater reduction than SR02 alone. Although the reduction in *E. coli* UTI89 invasion of the cocktail on UM-UC-3 cells was observed lowest compared to the others, it did not significantly differ from the pre-treatment with SR04 alone.

We next examined the effect of phages SR02, SR04, or the cocktail on the expression of proinflammatory cytokine genes in UM-UC-3 cells under both uninfected and infected conditions. In the uninfected condition (Figs. 6b-f; uninfected), phage treatment did not induce the expression of any proinflammatory cytokine genes, indicating that the phage treatment did not harm UM-UC-3 cells. Once *E. coli* UTI89 infection initiated for an hour, we observed some changes of gene expression pattern. Our data showed no increases in the proinflammatory cytokine genes *TNF-a* and *IL-6* in any groups (Figs. 6b and d, respectively) and only a slight increase in *IL-1b* expression was observed (Fig. 6c). Interestingly, *E. coli* UTI89-infected UM-UC-3 cells pre-treated with either SR04 or the cocktail exhibited substantially increases in the expression of *MIP- 3* and *IL-8* (Figs. 6e and f, respectively).

## Discussion

The threat of multidrug-resistant (MDR) bacteria poses significant challenges to patients, healthcare systems, and economies worldwide^55^. MDR-UPEC is one of the most problematic issues that requires careful antibiotic selection or the development of new therapies^56^. Phage therapy, which was once overshadowed by the discovery of easy-made, broad-spectrum antibiotics, has re-gained interest as a promising therapeutic alternative due to its distinct resistance mechanism and minimal side effects^28,57^. Despite successful cases of phage therapy, similar to antibiotics, the use of phages would lead to phage resistance that might occur during phage-bacterial arms race^28,58,59^. One promising approach to overcome the phage resistance in bacteria is to formulate phage cocktails that are composed of genetically diverse phages since multiple mutations in bacteria to fend off the phage infection are barely occurred^28^. Here, we rapidly screened and selected promising phage candidates that we first assumed they are genetically divergent from our phage library using killing assays and lysogeny tests. Through this screening, we easily eliminated inappropriate phages capable of integrating their genomes into the host genome. Notably, among the phages tested, SR02 exhibited low efficacy in suppressing *E. coli* UTI89 growth (Fig. 1b) and was expected to have little to no impact on enhancing the overall efficiency of the phage cocktail. However, when combined with the highly effective SR04, the cocktail produced a surprising outcome, significantly enhancing bacterial suppression and substantially delaying bacterial growth (Fig. 1c). This synergistic interaction between SR02 and SR04 prompted further investigation into their biological characteristics, evolutionary relationship, and potential therapeutic applications.

Highly efficient phages, characterized by rapid adsorption, short latent periods, and large burst sizes, are recommended and highly preferable for phage therapy^60,61^. Our novel phages, SR02 and SR04, possess some characteristics that align with these criteria for highly efficient phage selection. Both exhibited rapid adsorption, occurring within approximately 5 min post-infection (Fig. 2c). SR04, in particular, had a relatively short latent period (20 min) and produced a large burst size (Fig. 2d), conferring a competitive advantage over its bacterial host. As expected, SR04 treatment resulted in a prominent outcome in bacterial growth suppression during the initial 5–6 h (Fig. 2f), regardless of the MOIs used; however, phage-resistant bacteria rapidly emerged afterwards. The resistance mechanism is possibly mediated by receptor adaptation of the bacterial host, host defense systems, or phage-derived defense systems, allowing a small population of phage-resistant mutants to diminish treatment efficacy, resulting in bacteria regrowth^62^. However, phage resistance is inevitable and has evolved during the evolutionary arms race to counter phage invasion, regardless of how sophisticated the replication machinery employed by the phages is^63–66^. A similar killing pattern has also been observed in previously discovered phages of the same genus *Kayfunavirus*^67–69^, suggesting common phage-host interactions among these phages. However, SR02 displayed a longer latent period and lower burst size compared to SR04 (Fig. 2d). It is not surprising that SR02 underperformed in comparison to SR04. The lysis efficacy of SR02 was poor, as it only suppressed bacterial growth for a short period of time and had no significant impact on overall bacterial growth (Fig. 2e). Previous studies on the phage, øEc_1, which also belongs to the genus *Kuravirus*^70^, and on phages Lu221 and Hi226, classified in the same order as the *Kuravirus*-like family^71^, demonstrated that cell density during treatment was maintained at levels comparable to untreated host strains. Interestingly, phages Lu221 and Hi226 are plasmid-dependent phages that recognize bacteria carrying conjugative plasmids^71^. Further investigation into the phage-host interplay contributing to the low efficacy of SR02 in treatment might be required. Moreover, since the phages SR02 and SR04 were enriched by UPEC strain UTI89 as a model, they tend to display restricted host spectrum. Our data revealed that they were both highly specific to UPEC strain UTI89 and exhibited inefficient activity against some clinical isolates. Further genomic analysis of these clinical isolates in comparison to our bacterial model is essential to identify their diversity that might play a role in phage sensitivity.

Remarkedly, compared to treatment with either single phage, the combination of phages SR02 and SR04 resulted in an effective phage cocktail that strongly suppressed bacterial growth and minimized the emergence of phage-resistant bacteria. One of the key strategies in formulating phage cocktails is to combine genetically divergent phages. The hypothesis is that resistance to one phage may not affect the ability of distantly-related phages to infect, and superinfection immunity is less likely to occur when co-infection involves unrelated phages^72^. Our recent study demonstrated that using two genetically diverse phages in a cocktail effectively suppressed the emergence of phage-resistant *P. aeruginosa*^28^. We formulated a cocktail by combining a small lytic phage with a nucleus-forming jumbophage that recognized different receptors for phage entry, thereby leading to high efficiency in both bactericidal activity and resistance delay. Additional support for the use of genetically diverse phages in cocktails comes from the study of Wandro et al., (2022), which found that some phages were ineffective when used individually but became more effective when combined in cocktails^73^. In our study, phages SR02 and SR04 exhibited substantial differences in their plaque and phage morphologies (Figs. 2a and b) and belonged to different families, based on genomic data (Figs. 3 and 4). The cocktail of these phages efficiently killed *E. coli* UTI89 and effectively suppressed the growth of phage-resistant bacteria, suggesting a reduced likelihood of bacterial evolution to overcome phage infection when challenged by a well-combined mixture of genetically diverse phages. Our findings also support the observation that cocktails composed of closely related phages exhibit lower efficiency. Phages SR04 and Zappy shared similar plaque morphology and RFLP patterns, indicating their close relationship (Supplementary Figs. 1 and 2), and their combination resulted in the same killing profile as when used alone (Fig. 1b and c). Therefore, the high efficacy of our phage combination of SR02 and SR04, as evidenced by the improved killing profile in this study, is attributed to the combination of distinctly related phages.

Due to the drastic change in bacterial killing patterns when phages SR02 and SR04 were combined (Figs. 1c and 5a), we suspect that, in addition to the combination of genetically diverse phages, coinfection with synergistic effects might be occurring during phage treatment. The mechanisms behind synergistic coinfection remain poorly understood. A phenomenon similar to our findings was recently demonstrated in a report in 2023 ^74^. Lauman and Dennis combined a poorly performing phage, described as a lysogenization-capable (LC) phage with a low frequency of stable lysogenization, with an effective killing phage in a cocktail. This combination maximized antibacterial outcomes through additive or synergistic effects of phage-phage interactions^74^. In our study, attachment sites (attL and attR), which are involved in the site-specific recombination system of lysogenic phages, were identified in the genome of SR02 (Supplementary Table S3). However, other characteristics from our current experiments did not support the lysogenic potential of SR02. A deeper understanding of the life cycle of SR02, including its potential capability to integrate its genome into the host chromosome, is necessary. The use of LC phage could reveal novel strategies for phage selection in cocktails. Moreover, intense studies into how SR02 and SR04 exert their synergistic effects will enhance our understanding of this phage cocktail’s potential for therapeutic use.

In our study using a cell culture model, we compared the efficacy of single phages and their combinations. Our data demonstrated that pre-treatment with phages SR02, SR04, and the phage cocktail reduced the invasiveness of *E. coli* UTI89 to human bladder epithelium cells, UM-UC-3, with the cocktail achieving the highest reduction in the number of surviving bacteria (Fig. 6a). These data suggest that it would be difficult for *E. coli* UTI89 to develop resistance to the phage combination via multiple mutations and overcome their killing capacity. Surprisingly, phage SR02, which was less efficient in killing *E. coli* UTI89 in vitro, as demonstrated by the killing assay (Fig. 2e), significantly decreased the invasive properties of the bacteria in the in vitro cell culture model. This observation suggests that SR02 was able to alter the mechanisms of *E. coli* UTI89 infection, despite its low ability to kill the bacteria.

Phages are a predominant component of the gastrointestinal virome, also referred to as the gut phageome^75^, and are believed to be beneficial to humans by maintaining immune system balance and controlling pathogen proliferation in the human body^76^. Hence, we also investigated whether our phages serve a role in the immune response within a urinary-related cell culture model. In *E. coli* UTI89-uninfected UM-UC-3 cells, none of the phages triggered the expression of key proinflammatory cytokine genes (*TNF-α*, *IL-1b*, *IL-6*, *IL-8*, and *MIP-3*), indicating their low immunogenicity (Figs. 6b-f; uninfected). This is unsurprising and is consistent with previous studies showing that many phages and their proteins have low immunogenicity and do not strongly stimulate the adaptive immune response or reactive oxygen species (ROS) production both in vitro and in vivo^77,78^. However, the expression of cytokines and immune-related genes can vary depending on the phage. Some phages act as immune modulators due to certain components, such as the capsid protein of a T4 phage or the head of coliphages, which harbor a protein sequence that can stimulate the human immune system^79^. On the contrary, some studies reported reduced proinflammatory cytokine level after phage treatment. For instance, treatment of filamentous Pf phage lowered levels of neutrophil chemokine *CXCL1* and the proinflammatory cytokines *IL-1b* and *IL-17* in the bronchoalveolar lavage fluid from mice^80^. Another study using a phage cocktail showed down-regulation of *IL-1b*, *IL-6*, and *IL-8* in a zebrafish model, even in the absence of bacterial infection^81^. Similarly, phage Pf4 was able to reduce TNF-α production in mouse and human immune cells when lipopolysaccharide was administered alone^82^. Upon *E. coli* UTI89 infection in UM-UC-3 cells pre-treated with SR04 or the cocktail, *IL-8* and *MIP-3* expression was highly upregulated (Figs. 6e and f). *IL-8* and *MIP-3* are chemoattractant cytokines known for attracting and activating neutrophils and macrophages in inflammatory regions, respectively^83,84^. This may be due to the effective bacterial killing by SR04 and the cocktail, causing bacterial lysis and the release of immunostimulatory agents that trigger *MIP-3* and *IL-8* expression. To confirm the biological functions of these cytokines, further in vivo experiments in co-culture or animal models are needed. Interestingly, SR02 did not increase the expression of any cytokine genes, indicating strain-specific effects of bacteriophages on host immune responses. Given the altered gene expression patterns of cytokines, we suggest that the appropriate phage dosage should be carefully and individually optimized to prevent negative impacts on human cells and to avoid internal inflammation.

## Materials and methods

### Ethical approvals

This work was reviewed and approved by the Chulalongkorn University Institutional Biosafety Committee (CU-IBC) (Approval No. SC CU-IBC-028/2020 Ex1) in accordance with the risk levels of pathogens and animal toxins outlined in the Risk Group of Pathogen and Animal Toxin (2017) published by Department of Medical Sciences (Ministry of Public Health), the Pathogen and Animal Toxin Act (2015), and the Biosafety Guidelines for Modern Biotechnology BIOTEC (2016). Clinical uropathogenic *E. coli* isolates used in this study were obtained from urine culture of patient’s admitted to Maharaj Nakorn Chiang Mai Hospital (MNCMH) in 2020 (Approval No. 097/2567 by the Research Ethics Committee, Faculty of Medicine, Chiang Mai University).

### Phage preparation

Freshwater samples were collected from canals in Samut Prakan province and Bangkok, Thailand. Phages were enriched from 50 mL of freshwater by adding 5 mL of Luria-Bertani (LB) medium (Tryptone; Himedia™, Cat. No. RM027, and Yeast extract; Himedia™, Cat. No. RM014) ,250 µL of 100 mM CaCl_2_, and 1 mL of overnight cultured *E. coli* UTI89. The mixture was incubated at 30 °C with shaking at 200 rpm for 48 h^85^. The enriched phages were harvested by centrifugation at 4 °C, 9,000 rpm for 15 min and the supernatants were filtrated through a 0.45 μm filter. The phage lysate was stored at 4 °C for further experiments. For the phage purification, the full plate double-layer agar (DLA) method was employed. The phage lysate was serially diluted 10-fold with SM buffer. A 10 µL aliquot of each dilution was mixed with 100 µL of overnight cultured *E. coli* UTI89 and 5 mL of 0.35% molten LB top agar. The mixture was immediately poured onto the 1.5% LB bottom agar plate. The DLA plates were incubated overnight at 30 °C. Single plaques were picked and resuspended in 100 µL SM buffer. This purification step was repeated at least 3 times to obtain a single phage strain. To prepare a high titer of purified phages, the phages in SM buffers were diluted to an appropriate concentration, and the full plate DLA method was repeated. Plates exhibiting web lysis patterns (nearly confluent plaques) were soaked with 5 mL SM buffer at room temperature for at least 5 h^86^. The solutions were collected and centrifuged at 4 °C, 9,000 rpm for 5 min, and filtered through 0.45 μm filter. The high titer purified phages were stored at 4 °C until use. Phage titer was determined by performing a spot test using various 10-fold serial dilutions on DLA plates.

### Killing efficiency profile

The killing efficacy of phages against *E. coli* UTI89 was determined by monitoring the growth kinetics of the bacterial host. *E. coli* UTI89 in early mid-log phase (optical density at 600 nm (OD_600_) ≈ 0.3, corresponding to approximately 2.4 × 10^8^ CFU/mL) was mixed with an equal volume of phage-containing SM buffer at high titer or with the determined MOI values of 0.01, 0.1, 1, 10, and 100 in 96-well plates. For the phage cocktail formulation, two phages were prepared to obtain equal concentrations (phage number/mL) before being mixed in an equal ratio to achieve the desirable MOI. OD_600_ was immediately measured using a microplate reader (Synergy™ H1, Biotek), and subsequent readings were taken every 10 min for 16 h. The plates were incubated at 37 °C during the experiments. Each absorbance value was plotted against time to illustrate the bacterial growth kinetics.

### Viable bacterial cell and phage count by colony and plaque forming counting

The liquid culture at desired time points was serially diluted 10-fold in normal saline solution (NSS) and 3 µL of each dilution was spotted on LB agar plates. For phage counting, the same liquid culture was filtrated through 0.45 μm filters. The filtrates were then serially diluted 10-fold in SM buffer and 5 µL of each dilution was mixed with overnight cultured *E. coli* UTI89 and 0.35% molten LB top agar. The mixture was immediately poured onto the 1.5% LB bottom agar plate. The plates were incubated overnight at 37 °C and the resulting colonies or plaques were counted.

### Isolation, purification, and selection of phage-resistant *E. coli* UTI89

Full plate DLA was performed using high titer phage. After overnight incubation, single colonies were picked and streaked onto LB plates. Phage-resistant isolates were re-streaked three times to confirm their identity. A single colony from each isolate was cultured overnight, and 50 µL of each phage-resistant *E. coli* UTI89 was mixed with 2 mL of 0.35% molten LB top agar, which was then immediately poured into 6-well plates. Once the agar had solidified, high titer phage was spotted onto the bacterial lawn. The plates were incubated overnight. Isolates that showed resistance to phage (no plaque observed) were used for the lysogeny test. All incubations were performed at 37 °C.

### Lysogeny test

Lysogens were confirmed by the release of phage following prophage induction by mitomycin C treatment. Each phage-resistant isolates in mid-log phase culture (OD_600_ ≈ 0.4) were treated with mitomycin C to achieve final concentration of 0.1–1 µg/mL. Bacteria had been continuously cultured for 2 h, after which the supernatant was collected by centrifugation at 12,000×g for 5 min and filtered through 0.45 μm filters. The filtrates were serially diluted and spotted onto the bacterial lawn of DLA plates. The plates were incubated overnight at 37 °C and plaques were observed if present. The *E. coli* strain UTI89 treated with mitomycin C was used as a negative control since it is not lysogenized by phages. High-titer lysates of phages were used as a reference control to visualize plaque morphology for the corresponding phage isolates.

### Host range and efficiency of plating analysis

The spot test was performed using 10-fold serial dilutions of phages to assess their infectivity against 13 different *E. coli* strains, including 2 laboratory strains, 1 probiotic strain, 3 UPEC strains, 6 clinical UPEC isolates, and 1 diarrheagenic *E. coli* strain, and also *Salmonella enterica* Typhimurium as indicated in Table 1. Briefly, 5 µL of each phage concentration was spotted onto the bacterial lawn of each strain and incubated overnight at 37 °C. The number of plaques produced in each bacterial strain was counted, and the efficiency of plating (EOP) was calculated as the average PFU on the target bacteria divided by the average PFU on host bacteria, based on triplicate experiments. EOP values were classified according to the efficiency of killing as follows: highly productive (≥ 0.5), medium productive (0.1–0.5), low productive (0.001-0.1), or inefficient (< 0.001)^87^.

### Transmission electron microscopy

Phage suspension was mixed with NaCl and polyethylene glycol (PEG) 8000 to achieve final concentrations of 1 M and 10%, respectively. After incubation overnight at 4 °C for precipitation, the suspension was centrifuged at 8,500 rpm at 4 °C for 10 min. The supernatant was discarded, and the pellet was dried before being resuspended in SM buffer without glycerol. Phage particles were observed under a transmission electron microscope (TEM) (HITASHI model HT7700) by negative staining with 2% uranyl acetate, as performed by Laboratory Services, Department of Tropical Pathology, Faculty of Tropical Medicine, Mahidol University.

### Adsorption assay

To determine the time required for phages to attach to their host, a mid-log phase culture of *E. coli* UTI89 (OD_600_ ≈ 0.4, corresponding to approximately 3.2 × 10^8^ CFU/mL) was infected with phage at an MOI 0.01 and incubated at 37 °C. At each desired time point, 100 µL of the infected culture was harvested and filtrated through a 0.45 μm filter. The filtrates were serially diluted in SM buffer and the number of free phages was determined by spot titer assay. The percentage of free phage was plotted against time of post-infection. The experiment was performed in triplicates.

### One-step growth curve

To evaluate the latent period and burst size of a phage, 1 mL *E. coli* UTI89 (OD_600_ ≈ 0.4) was infected with phage at MOI 0.01. The mixture was incubated at 37 °C for 5 min for phage absorption, then centrifuged at 9,000 rpm for 1 min to remove free phages. The supernatant was used to determine the number of unabsorbed phages, which was then subtracted from the initial phage number to calculate the number of infected cells. The pellet was washed in 1 mL of LB to remove any remaining unbound phages. After centrifugation, the pellet was resuspended in 20 mL of LB, and the cell suspension was incubated at 37 °C. At designated time points, 200 µL of cell suspension was collected and filtered through a 0.45 μm filter. The number of produced phages in the filtrates was then quantified by spot titer assay and converted to phage concentration (PFU/mL), which was plotted against time to generate a phage growth curve. The latent period was defined as the time interval between the start of the experiment to the onset of the phage production. The burst size was calculated by subtracting the number of unabsorbed phage from the average maximum phage yield and dividing the difference by the number of infected cells (PFU/CFU)^88^.

### Analysis of pH and temperature tolerances

To assess phage viability under pH stress, 100 µl of phage lysate was mixed with 900 µl of SM buffer at various pH (2, 4, 6, 7.5, 8, and 10) and incubated at 37 °C for 1 h. For temperature tolerance, 50 µl of phage lysate was incubated for 1 h at different temperatures (4, 20, 25, 30, 37, 40, 50, 60, and 70 °C). In both tests, the titers of surviving phages were determined by spot titer assay. The experiment was performed in triplicates.

### Phage DNA extraction

High titer phage lysates were concentrated by PEG precipitation. The lysate was mixed with NaCl and PEG8000 to achieve final concentrations at 1 M and 10% w/v, respectively. After overnight incubation at 4 °C, the mixture was centrifuged at 4 °C, 9,000 rpm for 10 min. The supernatant was discarded and the pellet was air dried before being resuspended in SM buffer. To eliminate bacterial genetic material contamination, purified phage was treated with 10U DNase I and 0.1 mg/mL RNase A. The mixture was incubated overnight at 37 °C. Next, the phage capsid was digested with 0.5 mg/mL of proteinase K, 0.5% sodium dodecyl sulfate (SDS), and 20 mM EDTA, and incubated at 55 °C for 1 h. Phage DNA was extracted using the phenol-chloroform-isoamyl alcohol method. Briefly, an equal volume of phenol-chloroform-isoamyl alcohol (25:24:1) (Sigma Aldrich, Switzerland) was added to the capsid-broken phage solution, and the mixture was vigorously vortexed. The solution was then centrifuged at 15,000 × g for 5 min, and the top aqueous layer was transferred to a new microcentrifuge tube. The phenol-chloroform-isoamyl alcohol extraction step was repeated and the top aqueous layers were pooled. The DNA solution was treated with 1/10 volume of 3 M CH_3_CooNa and 2 volumes of cold absolute ethanol. The solution was gently mixed and precipitated at -20 °C for 2 h, followed by centrifugation at 21,000 × g for 20 min. The supernatant was removed, and the DNA pellet was washed with 70% ethanol and centrifuged at 21,000 × g for 10 min. After discarding the supernatant, the DNA pellet was air-dried until it appeared clear. The DNA was dissolved in TE buffer and stored at -20 °C^89^. DNA concentration and purity were assessed using NanoDrop 2000 spectrophotometer (Thermo Scientific).

### Whole genome sequencing and analysis

Phage genomic DNA was sequenced using Illumina MiSeq platform. The sequencing reads were assembled in Galaxy Europe (https://usegalaxy.eu/) using SPAdes, and contigs were further assembled with Cap3. The complete phage genome sequence was annotated using the RAST pipeline^90^. Structural open reading frames (ORFs) were identified with Prokka. All ORFs were functionally annotated using DNA master version 5.0.2 (https://phagesdb.org/DNAMaster/) with reference to the NCBI database. The phage genome map was constructed and visualized using Artemis: DNA plotter version 18.1.0 (https://sanger-pathogens.github.io/Artemis/Artemis/). Antimicrobial-resistant genes were predicted using ResFinder^91^, while putative toxin genes were identified via VirulenceFinder^92^.

### Phylogenetic tree construction

The phage genomes were blasted in NCBI and highly similar phages were selected for phylogenetic tree construction. Phages from different families or genera were also chosen for clustering purposes. The analysis was performed using the VICTOR web service (https://victor.dsmz.de/*)*, which is designed for genome-based phylogeny and classification of prokaryotic viruses^93^. Pairwise comparisons of nucleotide sequences were conducted using the Genome-BLAST Distance Phylogeny (GBDP) method^94^, following settings recommended for prokaryotic viruses^93^. The resulting intergenomic distances were used to infer a balanced minimum evolution tree, with 100 pseudo-bootstrap replicates of the branches. To confirm the novelty of our phages, pairwise intergenomic similarities were calculated using the Virus Intergenomic Distance Calculator (VIRIDIC)^51^. Genome organization comparisons between our phages and the most similar phages were visualized using Easyfig software version 2.1 (https://mjsull.github.io/Easyfig/files.html)^95^.

### Cell invasion with gentamicin protection assay in human bladder urothelium

The human bladder epithelium UM-UC-3 (ATCC CRL-1749) was cultured in a 24-well plate at 37 °C with 5% CO_2_ in a humidified incubator until the cell density reached approximately 10^5^ cells per well. The complete growth medium consisted of RPMI-1640 with 2.05 mM L-glutamine (Cytiva, Utah, USA), 10% fetal bovine serum (FBS) (Cytiva, Austria), and 1% penicillin/streptomycin (Cytiva, Austria). The cells were then synchronized by replacing the complete growth medium with RPMI-1640 lacking FBS and antibiotics for 24 h. Each well was pretreated with 10 µL of high titer phages SR02, SR04, or a combination of both (total 10^6^ PFU/well), or with SM buffer as a control, and incubated for 5 minutes. Following pretreatment, the phage-pretreated UM-UC-3 cells were infected with UTI89 at MOI of 20 and incubated at 37 °C for 1 h. Each well was washed with Dulbecco’s phosphate-buffered saline (DPBS) (Hyclone, Singapore) and incubated with 0.5 mL medium containing 100 µg/mL gentamycin sulfate solution (AppliChem, Germany) for 90 min. Finally, the cells were lysed with 0.5 mL of 1% Triton-X-100 (Thermo Fisher Scientific, USA), and intracellular UTI89 numbers were enumerated using a serial 10-fold plating technique on the appropriate LB agar.

### Detection of UM-UC-3 cell gene expressions by a quantitative polymerase chain reaction (qPCR)

To collect RNA from the infected UM-UC-3 cells, the cells were cultured in 6-well plates at a density of 10^6^ cells per well, pretreated with phages, and infected with *E. coli* UTI89 under the same conditions as described in the cell invasion assay. Subsequently, 1 mL of Trizol reagent (Ambion, USA) was added into each well for RNA extraction, and cDNA was synthesized by the RevertAid First Strand cDNA reagent (Thermo Fisher Scientific, Lithuania). qPCR was performed using a SYBR-Green-based real-time PCR reagent (Bioline, Tennessee, US) on a ViiA7 Real-Time PCR machine (Applied Biosystems, US), as previously described^88^. The relative fold change in mRNA expressions was calculated using the comparative Ct method (2^−ΔΔCT^), with *GAPDH* used as a housekeeping gene. The primer pairs used in this study are listed in **Supplementary Table S5**.

### Statistical analysis

Experiments were performed in triplicates and the data were analyzed using one-way ANOVA followed by Tukey’s HSD *post hoc* test, with a significance level of *P* ≤ 0.05.

## Electronic supplementary material

Below is the link to the electronic supplementary material.

Supplementary Material 1

## Data Availability

The nucleotide sequences of phages SR02 and SR04 were deposited in Genbank database, accession numbers: OQ870566 and OQ870567, respectively.
