# Supplementary material for: Rapid formulation of a genetically diverse phage cocktail targeting uropathogenic Escherichia coli infections using the UTI89 model
Source: Sci Rep. 2025 Apr 14;15:12832. doi: 10.1038/s41598-025-96561-y (PMC11997193; doi:10.1038/s41598-025-96561-y)
Supplement: Supplementary file 1 — Supplementary Material 1 [file 41598_2025_96561_MOESM1_ESM.pdf]

Supplementary materials

Rapid Formulation of a Genetically Diverse Phage Cocktail Targeting Uropathogenic *Escherichia coli* Infections Using the UTI89 Model

Pattida Kongsomboonchoke,[a] Panupon Mongkolkarvin,[b] Patiphan Khunti,[c] Jarukit Vijitphichiankul,[c] Poochit Nonejuie,[d] Parameth Thiennimitr,[b,e] Vorrapon Chaikeeratisak[c] *

[a] Biotechnology program, Faculty of Science, Chulalongkorn University, Bangkok, Thailand

[b] Department of Microbiology, Faculty of Medicine, Chiang Mai University, Chiang Mai, Thailand

[c] Department of Biochemistry, Faculty of Science, Chulalongkorn University, Bangkok, Thailand

[d] Center for Advanced Therapeutics, Institute of Molecular Biosciences, Mahidol University, Nakhon Pathom, Thailand

[e] Center of Excellence in Microbial Diversity and Sustainable Utilization, Chiang Mai University, Chiang Mai 50200, Thailand

Running title: Rapid Cocktail Formulation for UPEC

* Corresponding author: Vorrapon Chaikeeratisak (vorrapon.c@chula.ac.th)

Supplementary Material

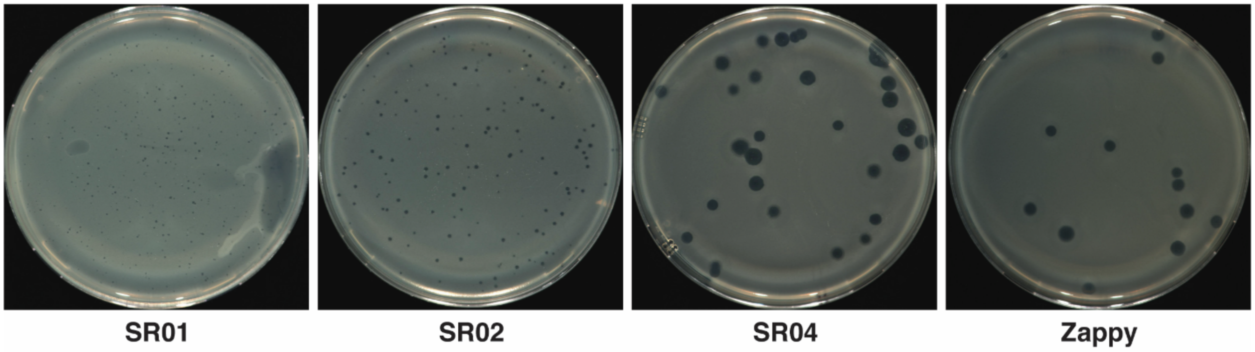

Figure S1. Plaque morphology of phages SR01, SR02, SR04, and Zappy. SR01 and SR02 produce small, turbid plaques while SR04 and Zappy produce large, clear, and smooth-edged plaques. An average diameter of SR01 was 0.17 ± 0.05 mm (mean ± SD, n = 30), while an average diameter of was Zappy 3.32 ± 0.62 mm (mean ± SD, n = 28).

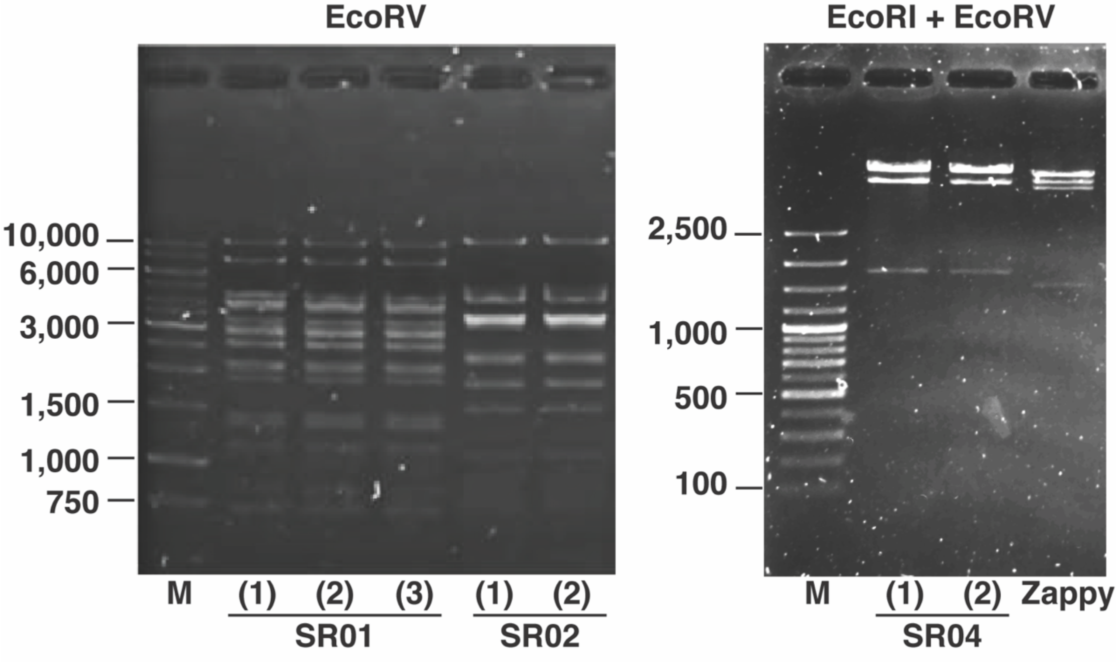

Figure S2. Restriction fragment length polymorphism (RFLP). The individual phage is classified based on plaque morphology displayed on DLA plates (Figure S1) and restriction fragment length polymorphism (RFLP) of those whose plaque morphology is indistinguishable.

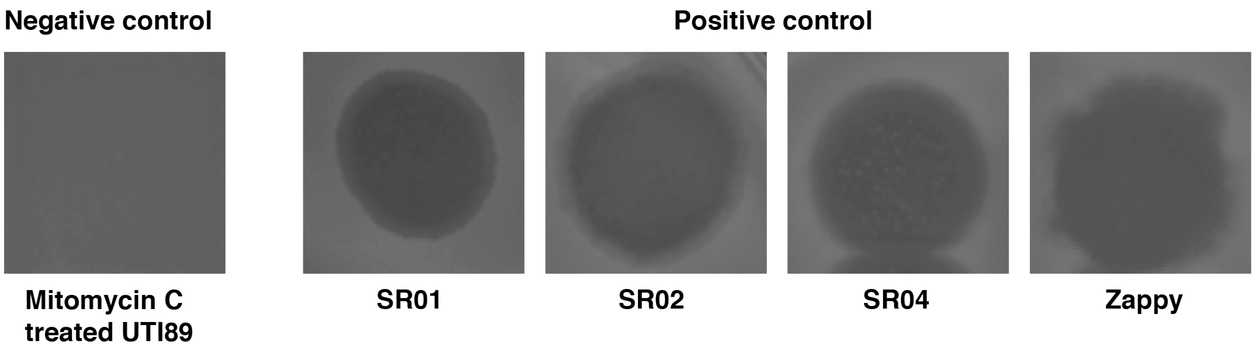

Figure S3. Lysogeny test. Supernatant of mitomycin C treated *E. coli* UTI89 is dropped on *E. coli* UTI89 lawn to show that the bacterial host is not lysogenized by any phages and represents as a negative control. Phage lysates (SR01, SR02, SR04, and Zappy) at high titer are dropped on bacterial lawn to demonstrate the ability to kill the bacterial host *E. coli* UTI89.

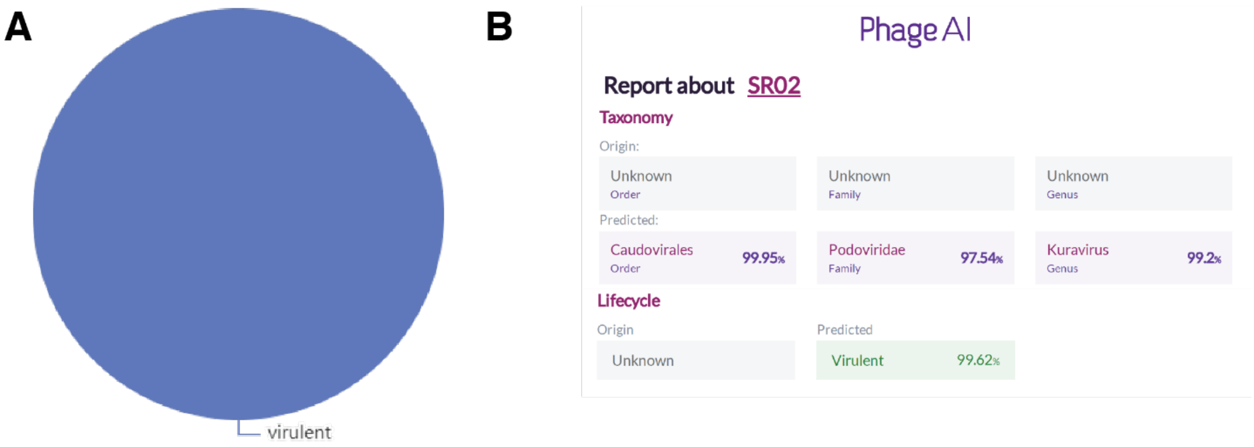

Figure S4. Prediction of phage SR02 life cycle. Both online tools PhageScope (A) and Phage AI (B) predict that phage SR02 is a virulent phage.

Table S1. Bacterial strains, serotypes, and fimtypes of *E. coli*.

**Bacterial strains**		**Serotypes and Fimtypes**
**Symptomatic UPEC strains**	*E. coli* UTI89	O18:H7:K1 [1]
	E. coli CFT073 (ATCC 700928)	O6:H1:K2 [2]<br>*fimH10* (analysis by FimTyper 1.0, https://cge.food.dtu.dk/services/FimTyper/, access date: 26/02/2025) [3]
**Asymptomatic UPEC strains**	*E. coli* ABU83972	O25:H1 (analysis by SerotypeFinder 2.0 (Software version: 2.0.1 (2020-07-27), Database version: 1.0.0 (2022-05-16))) [4]
**Commensal *E. coli* strains**	*E. coli* ATCC25922	O6:H1 (analysis by SerotypeFinder 2.0 (Software version: 2.0.1 (2020-07-27), Database version: 1.0.0 (2022-05-16))) [4]<br>*fimH30* (analysis by FimTyper 1.0, https://cge.food.dtu.dk/services/FimTyper/, access date: 26/02/2025) [3]
	E. coli MC4100	O16:H18 (analysis by SerotypeFinder 2.0 (Software version: 2.0.1 (2020-07-27), Database version: 1.0.0 (2022-05-16))) [4]
**Probiotic *E. coli* strains**	*E. coli* Nissle 1917	O6:H1:K5 [5]<br>*fimH30* (analysis by FimTyper 1.0, https://cge.food.dtu.dk/services/FimTyper/, access date: 26/02/2025) [3]
**Diarrheagenic *E. coli* strain**	Enterotoxigenic *E. coli* (ATCC 35401)	O78:H11:K80 [6]

***Salmonella enterica* Typhimurium**	Nalidixic acid-resistant derivative of ATCC 14028	

Table S2. Clinical isolates and the minimum inhibitory concentrations.

**Clinical samples**	**Minimum inhibitory concentrations (MICs, µg/mL), S: sensitive, I: intermediate, R: resistant<br>The drug susceptibility level is determined according to CLSI standards**												
	**Ampicillin**	**Amoxicillin-clavulanate**	**piperacillin/tazobactam**	**Ceftriaxone**	**Cefepime**	**Doripenem**	**Ertapenem**	**Imipenem**	**Meropenem**	**Amikacin**	**Gentamicin**	**Ciprofloxacin**	**Trimethoprim Sulfamethoxazole**
UPEC AT1	R≥32	R=16	R≥128	S≤1	S≤1	S≤0.12	S≤0.5	S≤0.25	S≤0.25	S≤2	R≥16	R≥4	S≤1
UPEC AT2	R≥32	R≥32	S=8	R=16	S≤1	S≤0.12	S≤0.5	S≤0.25	S≤0.25	S≤2	S≤1	R≥4	S≤1

UPEC AT3	R≥32	S=8	S≤4	S≤1	S≤1	S≤0.12	S≤0.5	S≤0.25	S≤0.25	S≤2	S≤1	S≤0.25	S≤1
UPEC AT4	R≥32	S=4	S≤4	S≤1	S≤1	S≤0.12	S≤0.5	S≤0.25	S≤0.25	S≤2	S≤1	S≤0.25	S≤1
UPEC AT5	R≥32	I=16	R≥128	R≥64	R≥64	S≤0.12	S≤0.5	S≤0.25	S≤0.25	S=16	S≤1	R≥4	R≥16
UPEC AT6	S=4	S≤2	S≤4	S≤1	S≤1	S≤0.12	S≤0.5	S≤0.25	S≤0.25	S≤2	S≤1	S≤0.25	S≤1

Table S3. List of annotated proteins from structural ORFs in the genome of phage SR02.

ORFs	Predicted function	Direction	Start	Stop	Size (n)	Sequence similarity	Accession no.	Database	E-value
ORF 1	Attachment site (attL)	+	1493	1504	12	-	-	PHASTER	0.0
ORF 2	putative tail fiber	-	1533	4175	2643	Escherichia phage phiEco32, complete genome	NC_010324.1	NCBI	0.0
ORF 3	Hypothetical protein	-	4185	4937	753	Phage vB_EcoP_SU10, complete genome	NC_027395.1	NCBI	0.0

ORF 4	Hypothetical protein	-	5037	6311	1275	Escherichia phage 172-1, complete genome	NC_028903.1	NCBI	3.85e-54
ORF 5	major head protein	-	6395	7456	1062	Phage vB_EcoP_SU10, complete genome	NC_027395.1	NCBI	0.0
ORF 6	scaffolding protein	-	7498	8583	1086	Phage vB_EcoP_SU10, complete genome	NC_027395.1	NCBI	0.0
ORF 7	Hypothetical protein	-	8583	8714	132	Phage vB_EcoP_SU10, complete genome	NC_027395.1	NCBI	3.20e-25
ORF 8	Portal protein	-	8829	11072	2244	Phage vB_EcoP_SU10, complete genome	NC_027395.1	NCBI	0.0
ORF 9	terminase large subunit	-	11134	12675	1542	Phage vB_EcoP_SU10, complete genome	NC_027395.1	NCBI	0.0
ORF 10	Hypothetical protein	-	12792	13265	474	Phage vB_EcoP_SU10, complete genome	NC_027395.1	NCBI	4.02e-110
ORF 11	Hypothetical protein	-	13322	13954	633	Phage vB_EcoP_SU10, complete genome	NC_027395.1	NCBI	2.61e-110

ORF 12	Hypothetical protein	-	13947	14201	255	Phage vB_EcoP_SU10, complete genome	NC_027395.1	NCBI	1.04e-50
ORF 13	Hypothetical protein	+	15286	16158	873	Phage vB_EcoP_SU10, complete genome	NC_027395.1	NCBI	0.0
ORF 14	Hypothetical protein	+	16185	16451	267	Phage vB_EcoP_SU10, complete genome	NC_027395.1	NCBI	5.45e-42
ORF 15	Hypothetical protein	+	16441	16689	249	Phage vB_EcoP_SU10, complete genome	NC_027395.1	NCBI	2.45e-49
ORF 16	Hypothetical protein	+	16800	17165	366	Phage vB_EcoP_SU10, complete genome	NC_027395.1	NCBI	4.21e-61
ORF 17	Hypothetical protein	+	19080	19979	900	Phage vB_EcoP_SU10, complete genome	NC_027395.1	NCBI	0.0
ORF 18	Hypothetical protein	+	20091	20309	219	Escherichia phage phiEco32, complete genome	NC_010324.1	NCBI	1.30E-35
ORF 19	Hypothetical protein	+	20306	20530	225	Enterobacteria phage NJ01, complete genome	NC_018835.1	NCBI	0.0
ORF 20	Hypothetical protein	+	20527	20736	210	Phage vB_EcoP_SU10, complete genome	NC_027395.1	NCBI	1.51e-39

ORF 21	Hypothetical protein	+	20806	21249	444	Phage vB_EcoP_SU10, complete genome	NC_027395.1	NCBI	6.23e-103
ORF 22	Hypothetical protein	+	21898	22287	390	Escherichia phage phiEco32, complete genome	NC_010324.1	NCBI	0.0
ORF 23	Hypothetical protein	+	22487	22783	297	Escherichia phage phiEco32, complete genome	NC_010324.1	NCBI	0.0
ORF 24	Hypothetical protein	+	22773	23006	234	Phage vB_EcoP_SU10, complete genome	NC_027395.1	NCBI	8.92e-31
ORF 25	Hypothetical protein	+	22996	23250	255	Phage vB_EcoP_SU10, complete genome	NC_027395.1	NCBI	1.51e-49
ORF 26	Hypothetical protein	+	23255	23470	216	Phage vB_EcoP_SU10, complete genome	NC_027395.1	NCBI	9.71e-27
ORF 27	Hypothetical protein	+	23829	23969	141	Phage vB_EcoP_SU10, complete genome	NC_027395.1	NCBI	1.07e-25
ORF 28	Hypothetical protein	+	24109	24285	177	Phage vB_EcoP_SU10, complete genome	NC_027395.1	NCBI	1.10e-34
ORF 29	Hypothetical protein	+	24282	24533	252	Phage vB_EcoP_SU10, complete genome	NC_027395.1	NCBI	6.62e-54

ORF 30	Hypothetical protein	+	24541	24705	165	Phage vB_EcoP_SU10, complete genome	NC_027395.1	NCBI	8.99e-27
ORF 31	Hypothetical protein	+	24929	25105	177	-	-	PHASTER	0.0
ORF 32	putative lipoprotein	+	25115	25414	300	Phage vB_EcoP_SU10, complete genome	NC_027395.1	NCBI	2.67e-45
ORF 33	Hypothetical protein	+	25416	25664	249	Phage vB_EcoP_SU10, complete genome	NC_027395.1	NCBI	6.57e-56
ORF 34	Hypothetical protein	+	25672	25893	222	Phage vB_EcoP_SU10, complete genome	NC_027395.1	NCBI	1.70e-24
ORF 35	Hypothetical protein	+	25903	26076	174	Phage vB_EcoP_SU10, complete genome	NC_027395.1	NCBI	4.89e-29
ORF 36	Hypothetical protein	+	26060	26293	234	Escherichia phage ES17, complete genome	MN508615.2	NCBI	1.80E-09
ORF 37	Hypothetical protein	+	26653	26964	312	Phage vB_EcoP_SU10, complete genome	NC_027395.1	NCBI	4.70e-70
ORF 38	Hypothetical protein	+	26973	27227	255	Phage vB_EcoP_SU10, complete genome	NC_027395.1	NCBI	3.81e-50

ORF 39	Hypothetical protein	+	27224	27469	246	Phage vB_EcoP_SU10, complete genome	NC_027395.1	NCBI	1.83e-31
ORF 40	Hypothetical protein	+	27475	27723	249	Phage vB_EcoP_SU10, complete genome	NC_027395.1	NCBI	2.46e-44
ORF 41	Hypothetical protein	+	27732	27848	117	Phage vB_EcoP_SU10, complete genome	NC_027395.1	NCBI	5.18e-21
ORF 42	Hypothetical protein	+	27955	28188	234	Phage vB_EcoP_SU10, complete genome	NC_027395.1	NCBI	8.58e-49
ORF 43	Hypothetical protein	+	28188	28454	267	-	-	PHASTER	0.0
ORF 44	Hypothetical protein	+	28454	28918	465	Phage vB_EcoP_SU10, complete genome	NC_027395.1	NCBI	5.51e-80
ORF 45	Hypothetical protein	+	28918	29133	216	Escherichia phage phiEco32, complete genome	NC_010324.1	NCBI	5.35e-45
ORF 46	Hypothetical protein	+	29369	29521	153	Phage vB_EcoP_SU10, complete genome	NC_027395.1	NCBI	7.68e-29
ORF 47	Hypothetical protein	+	29524	29886	363	Phage vB_EcoP_SU10, complete genome	NC_027395.1	NCBI	1.18e-79

ORF 48	Hypothetical protein	+	29898	31097	1200	Phage vB_EcoP_SU10, complete genome	NC_027395.1	NCBI	0.0
ORF 49	Hypothetical protein	+	31108	32079	972	Phage vB_EcoP_SU10, complete genome	NC_027395.1	NCBI	0.0
ORF 50	Hypothetical protein	+	32072	32878	807	Phage vB_EcoP_SU10, complete genome	NC_027395.1	NCBI	0.0
ORF 51	Hypothetical protein	+	32888	33505	618	Phage vB_EcoP_SU10, complete genome	NC_027395.1	NCBI	2.90e-149
ORF 52	putative amidoligase enzyme	+	33502	34605	1104	Phage vB_EcoP_SU10, complete genome	NC_027395.1	NCBI	0.0
ORF 53	glutamine amidotransferase	+	34763	36715	1953	Phage vB_EcoP_SU10, complete genome	NC_027395.1	NCBI	0.0
ORF 54	Hypothetical protein	+	36718	36951	234	Escherichia phage phiEco32, complete genome	NC_010324.1	NCBI	0.0
ORF 55	Hypothetical protein	+	36932	37156	225	Phage vB_EcoP_SU10, complete genome	NC_027395.1	NCBI	4.17e-48

ORF 56	ATP-grasp enzyme	+	37159	38355	1197	Phage vB_EcoP_SU10, complete genome	NC_027395.1	NCBI	0.0
ORF 57	Hypothetical protein	+	38368	38610	243	Phage vB_EcoP_SU10, complete genome	NC_027395.1	NCBI	7.59e-53
ORF 58	Hypothetical protein	+	38607	38813	207	Phage vB_EcoP_SU10, complete genome	NC_027395.1	NCBI	1.26e-17
ORF 59	YtfP/UPF0131-like protein	+	38883	39302	420	Phage vB_EcoP_SU10, complete genome	NC_027395.1	NCBI	9.43e-99
ORF 60	primase/helicase	+	39522	41312	1791	Phage vB_EcoP_SU10, complete genome	NC_027395.1	NCBI	0.0
ORF 61	DNA polymerase	+	41306	41860	555	Phage vB_EcoP_SU10, complete genome	NC_027395.1	NCBI	3.34e-124
ORF 62	nucleotidyltransferase domain-containing protein	+	41862	43160	1299	Phage vB_EcoP_SU10, complete genome	NC_027395.1	NCBI	0.0

ORF 63	dCTP deaminase	+	43170	43724	555	Cronobacter phage vB_CsaP_GAP52, complete genome	NC_019402.1	NCBI	9.70e-89
ORF 64	capsid maturation protease	+	43708	44217	510	Salmonella phage vB_SenS_SB13, complete genome	NC_048781.1	NCBI	7.31e-18
ORF 65	Hypothetical protein	+	44218	44412	195	Phage vB_EcoP_SU10, complete genome	NC_027395.1	NCBI	1.72e-33
ORF 66	Hypothetical protein	+	44412	44621	210	Phage vB_EcoP_SU10, complete genome	NC_027395.1	NCBI	1.13e-44
ORF 67	Hypothetical protein	+	44694	45023	330	Phage vB_EcoP_SU10, complete genome	NC_027395.1	NCBI	7.31e-77
ORF 68	DNA-binding protein	+	45016	45579	564	Phage vB_EcoP_SU10, complete genome	NC_027395.1	NCBI	1.07e-136
ORF 69	Hypothetical protein	+	45641	45820	180	Phage vB_EcoP_SU10, complete genome	NC_027395.1	NCBI	2.89e-34
ORF 70	Hypothetical protein	+	45830	46030	201	-	-	PHASTER	0.0

ORF 71	thiol-disulfide isomerase and thioredoxin	+	46039	46314	276	Phage vB_EcoP_SU10, complete genome	NC_027395.1	NCBI	9.33e-62
ORF 72	thymidylate synthase thyX/thy1	+	46330	46986	657	Phage vB_EcoP_SU10, complete genome	NC_027395.1	NCBI	7.78e-161
ORF 73	putative NAD+ diphosphatase	+	46953	47120	168	Phage vB_EcoP_SU10, complete genome	NC_027395.1	NCBI	3.20E-22
ORF 74	Hypothetical protein	+	47175	47366	192	Phage vB_EcoP_SU10, complete genome	NC_027395.1	NCBI	1.58e-33
ORF 75	NAD-dependent DNA ligase	+	47529	47852	324	Phage vB_EcoP_SU10, complete genome	NC_027395.1	NCBI	1.72e-73

ORF 76	isoleucyl-tRNA synthetase	+	47849	48049	201	Enterobacteria phage NJ01, complete genome	NC_018835.1	NCBI	3.91e-41
ORF 77	PhoH-like protein	+	48228	48965	738	Phage vB_EcoP_SU10, complete genome	NC_027395.1	NCBI	0.0
ORF 78	Hypothetical protein	+	49022	49339	318	Phage vB_EcoP_SU10, complete genome	NC_027395.1	NCBI	1.70e-62
ORF 79	HNH endonuclease	+	49329	49679	351	Shigella phage SGF2, complete genome	MN148435.1	NCBI	0.0
ORF 80	Hypothetical protein	+	49839	50132	294	Phage vB_EcoP_SU10, complete genome	NC_027395.1	NCBI	0.0
ORF 81	Hypothetical protein	+	50202	50456	255	Phage vB_EcoP_SU10, complete genome	NC_027395.1	NCBI	7.62e-53
ORF 82	Hypothetical protein	+	50458	50742	285	Phage vB_EcoP_SU10, complete genome	NC_027395.1	NCBI	1.86e-55
ORF 83	Hypothetical protein	+	50746	50904	159	Escherichia phage phiEco32, complete genome	NC_010324.1	NCBI	4.92e-28

ORF 84	Hypothetical protein	+	50906	51310	405	Phage vB_EcoP_SU10, complete genome	NC_027395.1	NCBI	4.85e-76
ORF 85	DNA polymerase	+	51327	53171	1845	Phage vB_EcoP_SU10, complete genome	NC_027395.1	NCBI	0.0
ORF 86	Hypothetical protein	+	53237	53545	309	Phage vB_EcoP_SU10, complete genome	NC_027395.1	NCBI	2.56e-56
ORF 87	Hypothetical protein	+	53545	53736	192	Phage vB_EcoP_SU10, complete genome	NC_027395.1	NCBI	6.05e-38
ORF 88	Hypothetical protein	+	53801	53920	120	Escherichia phage phiEco32, complete genome	NC_010324.1	NCBI	2.70E-19
ORF 89	Hypothetical protein	+	53920	54087	168	Phage vB_EcoP_SU10, complete genome	NC_027395.1	NCBI	3.41e-33
ORF 90	Hypothetical protein	+	54097	54273	177	Phage vB_EcoP_SU10, complete genome	NC_027395.1	NCBI	3.14e-32
ORF 91	Hypothetical protein	+	54320	54712	393	Phage vB_EcoP_SU10, complete genome	NC_027395.1	NCBI	8.75e-90
ORF 92	Hypothetical protein	+	54709	54885	177	Phage vB_EcoP_SU10, complete genome	NC_027395.1	NCBI	1.90e-35

ORF 93	Hypothetical protein	+	54882	55049	168	Phage vB_EcoP_SU10, complete genome	NC_027395.1	NCBI	3.75e-32
ORF 94	Hypothetical protein	+	55046	55189	144	Phage vB_EcoP_SU10, complete genome	NC_027395.1	NCBI	2.42e-25
ORF 95	Hypothetical protein	+	55205	55420	216	Phage vB_EcoP_SU10, complete genome	NC_027395.1	NCBI	7.46e-43
ORF 96	putative serine/threonine protein phosphatase	+	55430	55933	504	Phage vB_EcoP_SU10, complete genome	NC_027395.1	NCBI	2.65e-120
ORF 97	Hypothetical protein	+	55899	56063	165	Phage vB_EcoP_SU10, complete genome	NC_027395.1	NCBI	7.69e-32
ORF 98	Hypothetical protein	+	56057	56164	108	Escherichia phage phiEco32, complete genome	NC_010324.1	NCBI	2.20E-11
ORF 99	Hypothetical protein	+	56471	57217	747	Phage vB_EcoP_SU10, complete genome	NC_027395.1	NCBI	0.0

ORF 100	Hypothetical protein	+	57275	57625	351	Phage vB_EcoP_SU10, complete genome	NC_027395.1	NCBI	1.18e-81
ORF 101	Hypothetical protein	+	57597	57770	174	Phage vB_EcoP_SU10, complete genome	NC_027395.1	NCBI	3.97e-34
ORF 102	Appr-1-p processing enzyme family protein	+	57770	58210	441	Phage vB_EcoP_SU10, complete genome	NC_027395.1	NCBI	0.0
ORF 103	RNA polymerase ECF sigma factor	+	58233	58877	645	Phage vB_EcoP_SU10, complete genome	NC_027395.1	NCBI	7.39e-159
ORF 104	putative GTP-binding protein	+	58874	59266	393	Phage vB_EcoP_SU10, complete genome	NC_027395.1	NCBI	1.04e-88

ORF 105	ATP-binding protein	+	59263	60156	894	Phage vB_EcoP_SU10, complete genome	NC_027395.1	NCBI	0.0
ORF 106	5'-3' exonuclease	+	60143	60961	819	Phage vB_EcoP_SU10, complete genome	NC_027395.1	NCBI	0.0
ORF 107	Hypothetical protein	+	60975	61154	180	Phage vB_EcoP_SU10, complete genome	NC_027395.1	NCBI	3.97e-37
ORF 108	Hypothetical protein	+	61141	61542	402	Phage vB_EcoP_SU10, complete genome	NC_027395.1	NCBI	2.29e-95
ORF 109	Hypothetical protein	+	61508	61762	255	Phage vB_EcoP_SU10, complete genome	NC_027395.1	NCBI	7.74e-57
ORF 110	Hypothetical protein	+	61771	61932	162	Phage vB_EcoP_SU10, complete genome	NC_027395.1	NCBI	2.98e-28
ORF 111	Hypothetical protein	-	62126	66547	4422	Phage vB_EcoP_SU10, complete genome	NC_027395.1	NCBI	0.0
ORF 112	Hypothetical protein	-	66615	68294	1680	Phage vB_EcoP_SU10, complete genome	NC_027395.1	NCBI	0.0

ORF 113	putative DNA injection protein	-	68308	69276	969	Phage vB_EcoP_SU10, complete genome	NC_027395.1	NCBI	0.0
ORF 114	Attachment site (attR)	+	69278	69289	12			PHASTER	
ORF 115	Hypothetical protein	-	69289	70341	1053	Phage vB_EcoP_SU10, complete genome	NC_027395.1	NCBI	0.0
ORF 116	internal virion protein	-	70361	71143	783	Phage vB_EcoP_SU10, complete genome	NC_027395.1	NCBI	0.0
ORF 117	Hypothetical protein	-	71153	72187	1035	Phage vB_EcoP_SU10, complete genome	NC_027395.1	NCBI	1.95e-152
ORF 118	putative tail tip fiber protein	-	72189	73217	1029	Phage vB_EcoP_SU10, complete genome	NC_027395.1	NCBI	0.0
ORF 119	surface protein	-	73201	76218	3018	Phage vB_EcoP_SU10, complete genome	NC_027395.1	NCBI	0.0

ORF 120	putative structural protein	-	76228	77031	804	Phage vB_EcoP_SU10, complete genome	NC_027395.1	NCBI	0.0
ORF 121	lysis protein	-	77044	77535	492	Phage vB_EcoP_SU10, complete genome	NC_027395.1	NCBI	4.61e-120
ORF 122	putative holin	-	77565	77783	219	Phage vB_EcoP_SU10, complete genome	NC_027395.1	NCBI	4.59e-45
ORF 123	putative tail protein	-	77876	78664	789	Phage vB_EcoP_SU10, complete genome	NC_027395.1	NCBI	1.82e-73

Table S4. List of annotated proteins from structural ORFs in the genome of phage SR04

ORFs	Predicted function	Direction	Start	Stop	Size (n)	Sequence similarity	Accession no.	Database	E-value
ORF 1	internal virion protein D	-	2827	6714	3888	internal virion protein D [Escherichia phage LM33_P1]	YP_009324517	NCBI	0.00E+00

ORF 2	internal virion protein	-	6720	9002	2283	internal virion protein [Escherichia phage vB_EcoP-U8]	QOI58476	NCBI	0.00E +00
ORF 3	internal virion protein B	-	9014	9601	588	internal virion protein B [Escherichia phage vB_EcoA_ASO2B]	UAW58426	NCBI	0.00E +00
ORF 4	internal virion protein	-	9586	10056	471	internal virion protein [Escherichia phage vB_EcoP_PR_Kaz2018]	QFP92952	NCBI	0.00E +00
ORF 5	non-contractile tail tubular protein	-	10133	12490	2358	non-contractile tail tubular protein [Escherichia phage vB_EcoP-101120B1-2]	QZI79804.1	NCBI	0.00E +00
ORF 6	tail tubular protein A	-	12490	13056	567	tail tubular protein A [Escherichia phage vB_EcoP_IMEP24]	UCR92077	NCBI	0.00E +00
ORF 7	hypothetical protein	-	13214	13420	207	hypothetical protein BOX15_gp36 [Escherichia phage LM33_P1]	YP_009324510	NCBI	7.90E-40

ORF 8	major capsid protein	-	13477	14526	1050	major capsid protein [Escherichia virus LS3]	QLF86369.1	NCBI	0.00E+00
ORF 9	capsid and scaffold protein	-	14653	15537	885	capsid and scaffold protein [Escherichia phage vB_EcoP-101136BS1]	QZI79917	NCBI	0.00E+00
ORF 10	portal protein	-	15634	17202	1569	portal protein [Escherichia phage Penshu1]	QEG09804	NCBI	0.00E+00
ORF 11	virion assembly protein	-	17214	17462	249	virion assembly protein [Escherichia phage vB_EcoP_F]	YP_009789110	NCBI	0.00E+00
ORF 12	hypothetical protein	-	17455	17823	369	hypothetical protein PE3_030 [Escherichia phage PE3-1]	YP_009044278	NCBI	0.00E+00
ORF 13	hypothetical protein	-	17864	18088	225	hypothetical protein [Escherichia phage TM1]	QNH91741.1	NCBI	2.40E-43

ORF 14	hypothetical protein	-	18103	18375	273	hypothetical protein ASO2B_029 [Escherichia phage vB_EcoA_ASO2B]	UAW58415.1	NCBI	0.00E+00
ORF 15	putative transmembrane domain containing protein	-	18512	18604	93	hypothetical protein Phage8_00036 [Escherichia phage vB_EcoP-U8]	QOI58491	NCBI	8.80E-14
ORF 16	exonuclease	-	18586	19455	870	exonuclease [Escherichia phage TM1]	USL94557	NCBI	0.00E+00
ORF 17	hypothetical protein	-	19445	19849	405	hypothetical protein [Enterobacteria phage IME177]	QYC97344	NCBI	0.00E+00
ORF 18	hypothetical protein	-	19891	20100	210	hypothetical protein [Escherichia phage Mt1B1_P3]	QNJ49149.1	NCBI	1.40E-43

ORF 19	hypothetical protein	-	20097	20381	285	hypothetical protein CLBP1_5.5 [Escherichia phage CLB_P1]	AGD81057	NCBI	7.10E-43
ORF 20	hypothetical protein	-	20381	20539	159	hypothetical protein [Escherichia phage CY1]	UOL49353	NCBI	3.90E-29
ORF 21	DNA polymerase	-	20539	22710	2172	DNA polymerase [Escherichia phage vB_EcoA_ASO2B]	UAW58411	NCBI	0.00E+00
ORF 22	hypothetical protein	-	22774	22938	165	hypothetical protein 101118UKE1_039 [Escherichia phage vB_EcoP-101118UKE1]	QZI79724	NCBI	3.30E-30
ORF 23	toprim domain-containing protein	-	22990	24690	1701	toprim domain-containing protein [Escherichia phage vB_EcoP_F]	YP_009789099	NCBI	0.00E+00
ORF 24	hypothetical protein	-	24701	24880	180	hypothetical protein F_27 [Escherichia phage vB_EcoP_F]	AQT25421.1	NCBI	6.40E-34
ORF 25	hypothetical protein	-	24949	25164	216	hypothetical protein HOR59_gp26 [Escherichia phage vB_EcoP_F]	YP_009789097	NCBI	1.60E-22

ORF 26	N-acetylmuramoyl-L-alanine amidase	-	25189	25650	462	N-acetylmuramoyl-L-alanine amidase [Escherichia phage vB_EcoP_F]	YP_009789096	NCBI	0.00E+00
ORF 27	hypothetical protein	-	25637	25861	225	hypothetical protein HOR59_gp24 [Escherichia phage vB_EcoP_F]	YP_009789095.1	NCBI	0.00E+00
ORF 28	endonuclease I	-	25845	26315	471	endonuclease I [Escherichia phage vB_EcoP_F]	YP_009789094	NCBI	0.00E+00
ORF 29	DUF2815 family protein	-	26315	27028	714	DUF2815 family protein [Escherichia phage vB_EcoP_F]	YP_009789093	NCBI	0.00E+00
ORF 30	host RNA polymerase inhibitor	-	27086	27244	159	host RNA polymerase inhibitor [Escherichia phage ZG49]	YP_009787269	NCBI	4.10E-28
ORF 31	hypothetical protein	-	27332	27559	228	hypothetical protein HOT77_gp16 [Kayfunavirus Vec13]	YP_009807339	NCBI	2.90E-44

ORF 32	nucleotide kinase	-	27549	27989	441	nucleotide kinase [Escherichia phage Mt1B1_P3]	QNJ49161	NCBI	0.00E +00
ORF 33	hypothetical protein	-	27982	28242	261	hypothetical protein [Escherichia phage CY1]	UOL49341	NCBI	0.00E +00
ORF 34	ATP-dependent DNA ligase	-	28744	29799	1056	ATP-dependent DNA ligase [Escherichia phage RDN8.1]	QID21008	NCBI	0.00E +00
ORF 35	dNTPase inhibitor	-	29799	30062	264	dNTPase inhibitor [Escherichia phage vB_EcoP_SP7]	QLF80645	NCBI	0.00E +00
ORF 36	hypothetical protein	-	30067	30246	180	hypothetical protein HOR59_gp16 [Escherichia phage vB_EcoP_F]	YP_009789 087	NCBI	2.00E-34
ORF 37	hypothetical protein	-	30415	30615	201	hypothetical protein HOR59_gp15 [Escherichia phage vB_EcoP_F]	YP_009789 086	NCBI	1.40E-15

ORF 38	DNA-dependent RNA polymerase	-	30634	33315	2682	DNA-dependent RNA polymerase [Escherichia phage vB_EcoP_F]	YP_009789085	NCBI	0.00E+00
ORF 39	gp0.38 protein	-	33413	33673	261	gp0.38 protein [Escherichia phage K1F]	YP_424915	NCBI	0.00E+00
ORF 40	hypothetical protein	-	33673	33870	198	hypothetical protein PE3_005 [Escherichia phage PE3-1]	YP_009044253	NCBI	6.80E-36
ORF 41	hypothetical protein	-	34022	34171	150	hypothetical protein ASO2B_004 [Escherichia phage vB_EcoA_ASO2B]	UAW58390.1	NCBI	1.20E-14
ORF 42	gp0.3 protein	-	34171	34506	336	gp0.3 protein [Escherichia phage K1F]	YP_424911.1	NCBI	0.00E+00
ORF 43	gp0.2 protein	-	34580	34825	246	gp0.2 protein [Escherichia phage K1F]	YP_424910.1	NCBI	1.50E-42

ORF 44	alpha trehalase	-	35043	35258	216	alpha trehalase [Escherichia phage TM1]	QNH91709	NCBI	5.60E-30
ORF 45	hypothetical protein	-	35857	36015	159	hypothetical protein ASO2B_048 [Escherichia phage vB_EcoA_ASO2B]	UAW58434.1	NCBI	1.20E-08
ORF 46	HNH endonuclease	-	36197	36739	543	HNH endonuclease [Escherichia phage PE3-1]	YP_009044296	NCBI	0.00E+00
ORF 47	terminase large subunit	-	36753	38519	1767	terminase large subunit [Escherichia phage vB_EcoP-22664UKE3-2]	QZI78582	NCBI	0.00E+00
ORF 48	HNH homing endonuclease	-	38512	38949	438	HNH homing endonuclease [Escherichia phage RDN8.1]	QID21046	NCBI	0.00E+00
ORF 49	endopeptidase	-	38949	39392	444	endopeptidase [Escherichia phage vB_EcoA_ASO2B]	UAW58432.1	NCBI	0.00E+00

ORF 50	DNA packaging protein small subunit	-	39494	39757	264	DNA packaging protein, small subunit [Escherichia phage vB_EcoA_ASO2B]	UAW58431.1	NCBI	0.00E+00
ORF 51	type II holin	-	39754	39951	198	type II holin [Escherichia phage YZ1]	YP_009798558	NCBI	1.60E-30
ORF 52	endo-N-acetylneuraminidase	-	40085	40471	387	endo-N-acetylneuraminidase [Escherichia phage vB_EcoP_PR_Kaz2018]	QFP92956	NCBI	0.00E+00

Table S5. Sequence of primer pairs used in this study.

Cell line	Target gene	primers
Human urothelium (UMUC-3)	IL-8	5'-GCCAACACAGAAATTATTGTAAAGCTT-3'<br>5'-CCTCTGCACCCAGTTTTCCTT-3'
	MIP-3α	5'-CTGCTTTGATGTCAGTGCTGCTAC-3'<br>5'-CTGCCGTGTGAAGCCCACAATAAA-3'

	IL-1β	5’-AAACAGATGAAGTGCTCCTTCCAGG-3’<br>5’-TGGAGAACACCACTTGTTGCTCCA-3’
	IL-6	5’-ATGAACTCCTTCTCCACAAGCGC-3’<br>5’-GAAGAGCCCTCAGGCTGGACTG-3’
	TNF-α	5’-CGGGACGTGGAGCTGGCCGAGGAG-3’<br>5’-CACCAGCTGGTTATCTCTCAGCTC-3’
	GAPDH	5’-CCAGGAAATGAGCTTGACAAAGT-3’<br>5’-CCCACTCCTCCACCTTTGAC-3’

References

1. Wright, K. J., Seed, P. C. & Hultgren, S. J. Uropathogenic *Escherichia coli* flagella aid in efficient urinary tract colonization. *Infect Immun* **73**, 7657–7668 (2005).
2. Luo, C., Hu, G.-Q. & Zhu, H. Genome reannotation of *Escherichia coli* CFT073 with new insights into virulence. *BMC Genomics* **10**, 552 (2009).
3. CGE Server. https://cge.food.dtu.dk/services/FimTyper/.
4. Joensen, K. G., Tetzschner, A. M. M., Iguchi, A., Aarestrup, F. M. & Scheutz, F. Rapid and Easy *In Silico* Serotyping of *Escherichia coli* Isolates by Use of Whole-Genome Sequencing Data. *J Clin Microbiol* **53**, 2410–2426 (2015).
5. Altenhoefer, A. *et al.* The probiotic *Escherichia coli* strain Nissle 1917 interferes with invasion of human intestinal epithelial cells by different enteroinvasive bacterial pathogens. *FEMS Immunol Med Microbiol* **40**, 223–229 (2004).
6. Roussel, C. *et al.* Spatial and temporal modulation of enterotoxigenic *E. coli* H10407 pathogenesis and interplay with microbiota in human gut models. *BMC Biology* **18**, 141 (2020).
